# The Mediterranean Diet, Its Microbiome Connections, and Cardiovascular Health: A Narrative Review

**DOI:** 10.3390/ijms25094942

**Published:** 2024-04-30

**Authors:** Vincenzo Abrignani, Andrea Salvo, Gaetano Pacinella, Antonino Tuttolomondo

**Affiliations:** 1 Internal Medicine and Stroke Care Ward, University of Palermo, 90127 Palermo, Italy; vincenzabri@gmail.com (V.A.); andrea.salvo996@gmail.com (A.S.); gaspare.parrinello@unipa.it (G.P.); 2 Department of Health Promotion, Mother and Child Care, Internal Medicine and Medical Specialties, University of Palermo, 90127 Palermo, Italy

**Keywords:** Western-type diet, Mediterranean-type diet, gut microbiota, short-chain fatty acid, trimethylamine N-oxide, bile acids, cardiovascular disease, atherosclerosis, nutrients

## Abstract

The Mediterranean diet (MD), rich in minimally processed plant foods and in monounsaturated fats but low in saturated fats, meat, and dairy products, represents one of the most studied diets for cardiovascular health. It has been shown, from both observational and randomized controlled trials, that MD reduces body weight, improves cardiovascular disease surrogates such as waist-to-hip ratios, lipids, and inflammation markers, and even prevents the development of fatal and nonfatal cardiovascular disease, diabetes, obesity, and other diseases. However, it is unclear whether it offers cardiovascular benefits from its individual components or as a whole. Furthermore, limitations in the methodology of studies and meta-analyses have raised some concerns over its potential cardiovascular benefits. MD is also associated with characteristic changes in the intestinal microbiota, mediated through its constituents. These include increased growth of species producing short-chain fatty acids, such as *Clostridium leptum* and *Eubacterium rectale*, increased growth of Bifidobacteria, Bacteroides, and *Faecalibacterium prausnitzii* species, and reduced growth of Firmicutes and *Blautia* species. Such changes are known to be favorably associated with inflammation, oxidative status, and overall metabolic health. This review will focus on the effects of MD on cardiovascular health through its action on gut microbiota.

## 1. Introduction

Numerous studies spanning several decades have demonstrated that adherence to the Mediterranean diet (MD) is associated with a reduced risk of cardiovascular disease [1], cancer [2], and enhanced cognitive health [3]. Broadly speaking, the MD represents the customary dietary pattern of populations residing along the Mediterranean Sea coast. Nevertheless, variations exist among the diets of the Mediterranean coastal countries, with the consistent element being the consumption of virgin olive oil.

Ancel Keys initially characterized the MD during the 1960s as a diet low in saturated fat and rich in vegetable oils, predominantly observed in regions like Greece and southern Italy [4].

The definitions encompass specific guidelines emphasizing a high consumption of extra virgin (cold-pressed) olive oil, vegetables, including leafy greens, fruits, cereals, nuts [5], and legumes, along with moderate intakes of fish, meats, dairy products, and red wine. Conversely, the MD encourages limited consumption of eggs and sweets. Each description provides recommendations regarding the frequency of consumption, such as “often”, “daily”, or “biweekly”, and offers subjective terms like “abundance”, “high”, “moderate”, “low”, “some”, and “vast” to indicate the quantities of these foods within the diet. Most descriptions do not provide specific numerical servings or serving sizes and do not specify the quantities of dietary additives, such as sauces, condiments, tea, coffee, salt, sugar, or honey. Some definitions do emphasize the consumption of predominantly wholegrain cereals [6,7].

Various types of diets, such as the Mediterranean, dietary approaches to stop hypertension (DASH), Western, vegetarian, ketogenic, and Paleolithic diets, have been subject to comparative studies regarding their impact on cardiovascular risk. For instance, plant-based diets have been associated with favorable outcomes such as reduced blood pressure, lower blood lipid levels, and decreased platelet aggregation compared to non-vegetarian diets. Additionally, they have demonstrated benefits in weight management, as well as a decreased risk of developing metabolic syndrome and type-2 diabetes [8]; however, strict adherence to such diets may entail potential risks, including hyperhomocysteinemia, protein deficiency, anemia, and decreased creatinine content in muscles [9]. Also, the ketogenic diet has been noted for inducing rapid and significant weight loss, alongside positive biomarker changes, such as reduced serum hemoglobin A1c levels in individuals with type-2 diabetes. Nonetheless, it is accompanied by a notable elevation in low-density lipoprotein cholesterol levels, leading many healthcare practitioners to exercise caution in endorsing it [10]. Also, the consumption of Western-style diets, characterized by high calorie intake, processed foods, and low nutritional quality, coupled with sedentary lifestyles, has led to a significant health concern termed metaflammation. This state of chronic metabolic inflammation is associated with the development of various non-communicable diseases (NCDs). Metaflammation plays a crucial role in the pathogenesis of several prevalent NCDs, including obesity, type-2 diabetes, cardiovascular diseases, certain cancers, and neurodegenerative disorders. Chronic low-grade inflammation is a hallmark of metaflammation, which arises from the continuous activation of the innate immune system due to excessive nutrient intake, particularly of fats and sugars, along with inadequate physical activity [11].

Surprisingly, the exact mechanisms of action are not yet fully understood and various hypotheses have been proposed to explain the potential beneficial effects of the MD. Among these, a presumed link between the MD and the intestinal microbiota was put forward no more than a few years ago; this idea is therefore considered relatively new [12].

Two human studies have now made progress toward better understanding the role of the gut microbiota and the MD in disease risk factors. The first one analyzed the effects on a population of healthy overweight or obese subjects with sedentary lifestyles who habitually consumed small amounts of fruits and vegetables during an 8-week follow-up [13]. The second, on the other hand, examined a population of elderly subjects considered non-frail or pre-frail [14]. Among the different food products offered to the MD group, there were less meat and refined grain products and more fish, fruit, vegetables, legumes and whole grains, as well as a daily portion of nuts. Therefore, this diet doubled the total amount of fiber, increased the ratio of plant to animal protein by 2.5 times, and included fewer saturated fatty acids and more polyunsaturated fatty acids [15]. In the second previously mentioned study, metabolomic analyses of stool, urine, and blood revealed a clear change after MD implementation, and this change was characterized by significant changes in several metabolomic biomarkers (e.g., higher urolithins, tryptophan betaine, and oxindole acid and lower 3-acetic and carnitine, p-cresol, and indoxyl sulphate) considered presumed signs of adherence to the MD. In addition to the metabolome, the authors found specific changes in the composition of the gut microbiota, such as a higher abundance of *Faecalibacterium prausnitzii* and *Roseburia* and a lower abundance of *Ruminococcus gnavus* and *R. torques*. Interestingly, the change in insulin resistance was linked to specific bacteria, and subjects who reduced their index of insulin resistance had higher baseline levels of *Bacteroides uniformis* and *B. vulgatus* and lower levels of *Prevotella* covers [14]. According to the authors, this is mainly due to the intake of fiber, some vitamins (C, B6, and B9), and various minerals. In contrast, in the control group, the changes were mostly related to a greater increase in total fat intake. Although, at baseline, there were already some differences in the composition of the gut microbiota between countries (mainly related to local dietary habits), the diversity was similar, and adherence to the MD was associated with an attenuated loss of microbiome diversity. Seventy-five operational taxonomic units (OTUs) provided high predictive performance for identifying microbiome response to the MD. Furthermore, 44 OTUs showed a positive association with diet adherence, i.e., they had a higher abundance when the MD was strictly observed, while 45 OTUs were negatively associated with diet adherence. The authors called these OTUs “diet positive” or “diet negative”. Diet-positive OTUs included *F. prausnitzii*, *Eubacterium rectale*, *Roseburia*, *Bacteroides thetaiotaomicron*, *P. copri*, and *Anaerostipes hadrus*. Diet-negative OTUs included *R. torques*, *Collinsella aerofaciens*, *Coprococcus came*, *Dorea formicigenerans*, *Clostridium ramosum*, *Veillonella dispar*, *Flavonifractor plautii*, and *Actinomyces lingnae*. It is important to note that these different taxa were shared between countries, reinforcing the fact that, despite different baselines and specific dietary habits between countries, MD drives the composition of the gut microbiota consistently [16].

From our research, it is evident that the Mediterranean diet, rich in fruits, vegetables, olive oil, and fish, reduces cardiovascular risk by promoting heart health. Its anti-inflammatory and antioxidant properties, combined with low saturated fat intake, contribute to lower incidences of cardiovascular diseases and improved overall cardiovascular well-being. This effect is also mediated by the positive alterations the diet induces in the gut microbiota, further enhancing its impact on cardiovascular risk reduction.

In this narrative review, the authors provide their expert insight; in addition, a literature search was undertaken on this topic in PubMed, Google Scholar, and clinicaltrials.gov to ensure relevant trials were discussed. Selection criteria of this manuscript included English language articles dealing with experimental and epidemiological and clinical aspects linking MD, microbiome, and CVD. This article, in addition, is based on previously conducted studies and does not contain novel data of animal and human study origin.

## 2. The Mediterranean Diet Components

The MD can be conceptually simplified through a pyramid representation. At the base of the pyramid, one finds the essential food items that should constitute the foundation of the diet, contributing the highest energy intake. As you ascend the pyramid, you encounter foods that should be consumed in moderate quantities, including those of animal origin and items rich in sugars and fats, which warrant moderation and are reserved for rare consumption for special events [7].

The most important foods of the MD are as follows [17]:

### 2.1. Extra-Virgin Olive Oil (EVOO)

EVOO serves as the primary source of unsaturated fatty acids and various constituents, including fat-soluble vitamins, polyphenols, chlorophylls, and phytosterols. The polyphenols found within olive oil exhibit a spectrum of beneficial properties, encompassing anti-inflammatory, antioxidant, neuroprotective, cardioprotective, anticancer, anti-obesity, anti-diabetic, antimicrobial, and antisteatotic effects. These effects are predominantly attributed to the presence of secoiridoid derivatives, notably oleuropein, oleacein, and oleocanthal, as well as simple phenolic compounds such as tyrosol and hydroxytyrosol [18]. Polyphenols may play a pivotal role in the acknowledged pharmacological attributes of olive oil, which encompass anti-atherogenic, antihepatotoxic, hypoglycemic, anti-inflammatory, antitumoral, antiviral, analgesic, purgative, and immunomodulatory activities. Furthermore, these polyphenols contribute to safeguarding against age-related neurodegenerative conditions [19]. Hence, the quality of EVOO is contingent not only upon the levels of free fatty acids stemming from triacylglycerol degradation (acidity) but also on its polyphenol content, the compounds accountable for its flavor profile, and a multitude of its health-promoting attributes [20]. Studies have indicated that hydroxytyrosol diminishes mitochondrial oxidative stress and neuroinflammation in Alzheimer’s disease (AD)-prone transgenic mice by triggering nuclear factor erythroid 2-related factor 2 (nrf2)-dependent gene expression [21], and EVOO polyphenols additionally boost Nrf-2 activation within the liver, resulting in the release of antioxidant enzymes [22]. Nrf2 is considered the principal regulator of redox homeostasis, and its activation inhibits pro-inflammatory mediators like a cytokines, cyclooxygenase-2 (COX-2), and nitric oxide synthase inducible (iNOS) [23]. Polyphenols present in EVOO mitigate inflammation by decreasing the expression and activity of transcription factors nuclear factor kappa-light-chain-enhancer of activated B cells (NF-κB) and activator protein 1 (AP-1). This effect is attributed to their capability to scavenge free radicals, break radical chains, and minimize the generation of reactive oxygen species (ROS) and reactive nitrogen species (RNS) [24]. Research findings have indicated that hydroxytyrosol demonstrates in vitro antimicrobial activity against several infectious agents in the gastrointestinal and respiratory tracts, including *Vibrio cholerae*, *Vibrio parahaemolyticus*, *Haemophilus influenzae*, *Salmonella typhi*, *Moraxella catarrhalis*, and *Staphylococcus aureus*. This antimicrobial effect is observed at relatively low inhibitory concentrations. Additionally, hydroxytyrosol exhibits antimicrobial properties against foodborne pathogens like *Listeria monocytogenes*, *Yersinia enterocolitica*, and *Salmonella enterica* [25].

### 2.2. Legumes, Cereals, and Nuts

The Mediterranean diet commonly incorporates legumes such as beans, lentils, and chickpeas [26]. These legumes are frequently combined with various cereals, fish, meats, and vegetables. Similarly, seeds and nuts like hazelnuts, almonds, tree nuts, and pistachios have been integral to the diet for millennia and are consumed on a daily basis. Nuts and legumes have been traditional dietary staples in the Mediterranean region, as well as in Asia and the Americas. The primary constituents of pulses and beans are flavanols [27], a category of polyphenols characterized by a ketone group in their chemical structure. These flavanols are associated with the reduction in endothelial dysfunction, the lowering of cholesterol and blood pressure, and the regulation of energy metabolism [28]. On a molecular level, a substantial portion of these effects is facilitated through interactions with nitric oxide metabolism in the endothelial cells lining the blood vessels. This interaction results in the amelioration of endothelial dysfunction, leading to enhanced vasodilation and reduced blood pressure. These biomarkers collectively serve as indicators of cardiovascular disease risk, thereby substantiating the protective influence of flavanols in the prevention of chronic cardiovascular conditions [29]. The germ of whole grains harbors a polyamine known as spermidine, which has demonstrated the capacity to extend the chronological lifespan in various organisms, including flies, nematodes, rodents, and human cells. Spermidine is recognized for its inhibition of histone acetyltransferases, thereby conferring greater resistance to oxidative stress, augmenting autophagy, and significantly diminishing subclinical inflammation and the occurrence of cell necrosis during the aging process [30].

### 2.3. Fruits and Vegetables

The Mediterranean climate provides an ideal environment for the cultivation of numerous vegetables and fruits that constitute a significant portion of the MD [31]. Indigenous Mediterranean vegetables encompass turnips, artichokes, lettuce, and radishes. Interactions with external regions have led to the introduction of novel varieties of fruits and vegetables. For instance, citrus fruits and eggplant were introduced from North Asia and India, while zucchini, tomatoes, potatoes, peppers, corn, and green beans were introduced to the Mediterranean region from the Americas. Epidemiological evidence demonstrates that dietary supplementation with fruits and vegetables rich in polyphenols offers benefits in both preventing and ameliorating the adverse effects of aging on neuronal communication and behavior [32].

Another prominent characteristic of the Mediterranean diet is its exceptionally elevated content of fiber, particularly insoluble fiber, with notable bioavailability. It has been demonstrated that the consumption of a high-fiber diet induces substantial alterations in the gut microbiota composition, both in rodents and humans, resulting in a reduction in Firmicutes and an increase in Bacteroidetes, notably *Bacteroides acidifaciens* [33]. This shift in microbiota composition yields heightened production of short-chain fatty acids, including acetate, propionate, and butyrate. Accumulating evidence from experimental animal studies suggests that the microbial generation of these short-chain fatty acids from dietary fiber exerts suppressive effects on the development of numerous inflammatory, autoimmune, and allergic diseases [34]. In accordance with the World Cancer Research Fund (WCRF), the consumption of vegetables has been associated with a decreased risk of developing cancers affecting the oral cavity, pharynx, larynx, esophagus, and stomach. However, the available evidence regarding the impact of vegetable consumption on the risk of cancers affecting the colorectum, endometrium, ovary, lung, and nasopharynx is limited [35]. The vegetables featured in the MD are abundant in various chemical compounds that exhibit potential benefits in the context of diverse cancer types. These compounds include lycopene in tomatoes, organosulfur compounds in onions and garlic, capsaicin in hot peppers, indol-3-carbinol, isothiocyanates, and sulforaphane in cruciferous vegetables, monoterpenes in oranges and lemons, polyacetylenes in pumpkin and carrots, spermidine and ferulic acid in whole grains, and ginkgetin in capers. Furthermore, the presence of estrogenic molecules with low potency, such as biochanin A, formononetin, daidzein, coumestans, and genistein in beans, enables them to compete with endogenous estrogens for binding to estrogen receptors, thereby obstructing their mitogenic effects [36].

### 2.4. Dairy Products

Traditionally, Mediterranean countries have exhibited low consumption of milk and dairy products, yet the ample availability of land dedicated to raising goats and sheep for their meat, milk, and wool has facilitated the production of yogurt, cheese, and other fermented dairy products. Milk serves as a rich source of lacto-fermented foods, including yogurt and cheese [37]. Lactose in milk undergoes fermentation to lactic acid through the action of lactic acid bacteria (LAB), specifically *Lactobacillus delbrueckii* subsp. *Bulgaricus* and *Streptococcus thermophilus*. This fermentation process results in a reduction in milk pH, thus thwarting the proliferation of pathogenic microorganisms [38]. The presence of bacteria in yogurt contributes to the transient microbiota and thereby enhances the gut environment. Several studies have indicated potential benefits of yogurt in the management of type-2 diabetes. For example, a meta-analysis of randomized controlled trials evaluating the use of yogurt in type-2 diabetes management reported a reduction in complications associated with regular consumption [39]. Another well-known fermented dairy product in the Mediterranean region is cheese, including varieties such as pecorino, halloumi, brie, chevre, manchego, feta, Parmigiano Reggiano, and ricotta. Consumption of cheese in modest amounts is recommended within the MD. During the initial stages of fermentation, LAB utilize milk carbohydrates, resulting in the production of indigestible oligosaccharides. The consumption of these oligosaccharides exerts prebiotic effects and enhances the beneficial gut microbiota [40]. Furthermore, the short-chain fatty acids generated through the metabolism of oligosaccharides and resistant starch, both prevalent in the Mediterranean diet, by the gut microbiota have the capacity to induce satiety by delaying gastric emptying. This process leads to an increase in the production of gut hormones, such as glucagon-like peptide-1 and peptide-YY. Importantly, the MD, in addition to promoting weight loss, has been associated with a significant reduction in fasting glucose, C-peptide levels, and free and total testosterone levels [41].

### 2.5. Fish

The Mediterranean region boasts a robust fishing tradition, resulting in substantial fish consumption. Polyunsaturated fatty acids (PUFAs) encompass long-chain ω-3 PUFAs, notably eicosapentaenoic acid (EPA, 20:5n-3) and docosahexaenoic acid (DHA, 22:6n-3), primarily obtained from fish and seafood, as well as alpha-linolenic acid, derived from various plant sources. Among PUFAs, ω-3 free fatty acids elicit anti-inflammatory effects via the generation of specialized pro-resolving mediators, known as oxylipins, through oxygenated metabolites [42]. A recent report from the American Heart Association has suggested that ω-3 PUFA supplements may decrease the risk of death resulting from coronary heart disease in patients with a history of coronary heart disease. This potential benefit may be attributed to a reduction in ischemia-induced sudden cardiac death. However, the treatment did not demonstrate a reduction in the occurrence of recurrent nonfatal myocardial infarction. Furthermore, the American Heart Association recommends a daily ω-3 supplement of 1 g for patients with cardiovascular disease. This supplementation is advocated for its potential advantages, including lowering triglyceride (TG) levels and the prevention of arrhythmias and atherosclerosis [43]. Nevertheless, environmental contaminants have hindered the potential benefits of omega-3 fatty acids. For example the presence of nanoplastics (NPs) have demonstrated the capacity to induce intestinal and neural toxicity in fish, with a particular focus on elucidating the role of neurotransmitter and intestinal microbiota interactions in the underlying mechanisms of toxicity. Alterations in 14 metabolites have been identified as correlated with changes in three major microbial groups: Proteobacteria, Firmicutes, and Bacteroidetes. These findings indicate that polystyrene nanoparticles (PS-NPs) elicit intestinal inflammation, growth inhibition, and hindered development in zebrafish, phenomena strongly associated with dysregulated regulation within the brain-intestine-microbiota axis [44]. Research has demonstrated that exposure to polystyrene nanoparticles (PS-NPs) induces oxidative stress, leading to inflammation and apoptosis in the heart of carp. Furthermore, the extent of damage was found to be inversely correlated with the particle size of PS-NPs [45].

### 2.6. Wine

In European Mediterranean countries, the MD is notably associated with the moderate consumption of wine during meals. Some studies have sought to identify biomarkers of polyphenol intake and specifically polyphenols derived from certain food groups, including those originating from red wine [46]. Researchers have observed that polyphenols present in the MD exert a direct influence on the process of autophagy; for instance, resveratrol, a polyphenol found in nuts, wine, and grapes, functions as an autophagy inducer [47]. The impact of resveratrol on autophagy may be elucidated by its capacity to enhance the activity of the deacetylase sirtuin 1, which subsequently regulates the activity of numerous autophagy-related proteins. Similarly, polyphenols found in virgin olive oil, such as oleocanthal and oleuropein, have been documented to promote autophagy [48]. Moreover, the connection between autophagy and atherosclerosis and cardiovascular diseases has been delineated [49]; for example, autophagy plays a pivotal role in the effective development and functioning of cardiomyocytes [50]. Furthermore, autophagy plays a fundamental role in regulating the inflammatory response of macrophages, potentially by constraining the activity of the inflammasome and the formation of foam cells, likely through the modulation of lipid turnover [51].

## 3. MD and Cardiovascular Outcomes: Clinical, Epidemiological, and Intervention Studies

The concept that higher adherence to the MD was associated with a lower cardiovascular disease (CVD) incidence and mortality was first proposed in the 1950s. From then, epidemiological studies in Italy [52,53], Greece [54,55,56,57], and Spain [58,59] and even in non-Mediterranean populations [60,61,62,63,64,65,66,67,68] showed benefits from long-term adhesion to the MD. In addition, randomized controlled intervention trials, like the secondary prevention trial, the Lyon diet heart study [69], the PREDIMED (PREvención con DIeta MEDiterránea) trial in a low-risk population [70], and others [71], reported associations with lower CVD. A recent Cochrane review update on 30 randomized controlled trials (12,461 participants) showed, in primary prevention, little or no effect of the PREDIMED intervention (advice to follow an MD plus supplemental extra-virgin olive oil or tree nuts) compared to a low-fat diet on CVD mortality (hazard ratio (HR) 0.81, 95% confidence interval (CI) 0.50–1.32) or total mortality (HR 1.0, 95% CI 0.81–1.24) over 4.8 years. There was, however, a reduction in the number of strokes (HR 0.60, 95% CI 0.45–0.80). For secondary prevention, in the Lyon diet heart study, there was low-quality evidence of a reduction in adjusted estimates for CVD mortality (HR 0.35, 95% CI 0.15–0.82) and total mortality (HR 0.44, 95% CI 0.21–0.92) [72].

Systematic reviews [73,74,75,76], meta-analyses [77,78,79,80], and umbrella reviews [81] confirmed the beneficial effects of the traditional MD on cardiovascular health, albeit with a moderate–high degree of inconsistency. This inverse association includes coronary heart disease (CHD), peripheral artery disease, chronic heart failure, sudden cardiac death, and ischemic stroke but apparently not hemorrhagic stroke [57,79,81].

The main findings of some of the most noticeable studies [52,53,54,55,56,57,59,60,61,62,63,65,66,67,68,69,70,71,73,74,75,76,78,79,80,81] evaluating the effects of adherence to MD on the prevention of cardiovascular diseases are summarized in the following Table 1.

Despite the relatively large number of studies, there is still some uncertainty regarding the effects of a Mediterranean-style diet on clinical endpoints and cardiovascular disease (CVD) risk factors for both primary and secondary prevention. These different effects might depend on the definition of MD, with a wide variety of dietary (i.e., MedDietScore) and adherence (i.e., PREDIMED test) indices. There are also challenges with isolating the MD from the typical Mediterranean lifestyle and culture (including prolonged ‘social’ meals and siestas).

As regards the effects of Mediterranean diet’s role in cardiovascular disease prevention in the developmental age, it is well known that nutrition can influence the body’s metabolic programming from early infancy [82]. Interest in the healthy-heart effects of MD in pediatric patients is increasingly growing, yet evidence is not as strong as that in adult subjects. Children’s increasing adherence to MD reduces incidence of metabolic syndrome, overweight, and obesity [83]. In addition, an obesogenic dietary pattern in childhood (7–10 years) is related to increased arterial stiffness, while Mediterranean-style and anti-inflammatory diets are related to decreased arterial stiffness and reduced cIMT in adolescence and in adulthood [84].

## 4. Favorable Mechanisms of MD on Cardiovascular Health

The exact biologic mechanism by which an increased adherence to the traditional MD exerts its beneficial effects on cardiovascular health is not known. However, accumulating evidence indicates that the most important adaptations induced by the MD pattern, high in antioxidants, micronutrients, flavonoids, nitrate, calcium, proteins, polyphenols, carotenoids, vitamins, and fiber but low in saturated/trans-fat and sodium, are as follows:

(a) Favorable effects on multiple specific cardiovascular risk factors [85,86] through a specific plasma metabolomic profile (mainly triglycerides and medium/long-chain acylcarnitines, amino acids, and steroids). MD improves insulin resistance, increases adiponectin concentrations [87], and decreases the hepatic fat content [88] with beneficial effects on diabetes mellitus and metabolic syndrome [89,90]. The lipid profile improves, too, with a decrease in plasma cholesterol [91], oxidized low-density lipoprotein [87,92], LDL-cholesterol, ApoB, and the ApoB/ApoA-I ratio and an increase in ApoA-I [93,94]. There are also putative favorable changes in the blood fatty acid profile, with increased levels of eicosapentaenoic and docosahexaenoic acid [95]. Then, both systolic and diastolic blood pressure decrease [94]. Finally, the MD-style diet may influence the potential negative relationship between elevated plasma ceramide concentrations and CVD [96];
(b) Modulating actions on sympathetic nervous system, reducing average heart rate [97] and heart rate variability, a measure of cardiac autonomic dysfunction [98];
(c) Protection against oxidative stress and inflammation. MD is associated with lower concentrations of inflammatory mediators, like C-reactive protein, interleukin-6, sICAM, P-selectin, and tumor necrosis factor-α [94], hallmarks of inflammaging, the peculiar low-grade, chronic, and “sterile” inflammatory state characterizing old age that represents a background pathogenetic mechanism linking metabolic risk factors to increased risk of chronic degenerative diseases [96,99,100]. The MD modulates the immune system, induces induction of detoxification enzymes [101], and has a low dietary inflammatory index [102] and is associated with lower intracellular reactive oxygen species production [103], an increase in serum markers of atheroma plaque stability, and a reduction in CD40 expression on monocyte surface [94]. Flavonoids, in particular, provide a variety of nutraceutical functions including antioxidant, antimicrobial, anti-inflammatory, antiangiogenic, antitumor, and improved pharmacokinetic properties [104]. The MD was also significantly associated with lower levels of subclinical gut inflammation, defined by fecal calprotectin [105] and higher concentrations of fecal short-chain acids (FSCAs) (propionate and butyrate) [106];
(d) Anti-atherosclerotic effects. Increase in endothelial progenitor cells [103,107] and endothelial-mediated nitric oxide (NO) synthesis leads to higher NO bioavailability [100,108] and consequent significant improvements in endothelial function [86], flow-mediated microvascular vasodilation [103,109], and arterial stiffness [110,111], as well as carotid intima-media thickness [112];
(e) Decrease in platelet aggregation and blood coagulation [87];
(f) Inhibition of nutrient sensing pathways by specific amino acid restriction [113];
(g) And, last but not least, gut microbiota-mediated production of beneficial metabolites [114].

## 5. Microbiota: Definitions and Functions

The surfaces of the human body are heavily populated by a highly diverse collection of bacteria, fungi, archaea, viruses, and protozoa, termed the microbiota. The largest and richest site is the gut (small and, mainly, large intestine), which harbors > 100 trillion microbial cells [115]. The microbiota and their genes, called the microbiome, have been studied intensely through the past years using novel metagenomic, metatranscriptomic, and metabolomic approaches [96]. Fecal microbiota composition and diversity may be evaluated by three methods: living organisms are determined using bacterial cultures, total DNA taxonomic composition is estimated by next-generation sequencing of the rRNA gene, and quantitative assessment of several taxa is performed using specific quantitative polymerase chain reaction (qPCR) [116]. Landmark microbiome–host genome-wide association studies have identified many SNPs associated with gut microbiota [117]. We distinguish α-diversity (e.g., number of microbes) and β-diversity (e.g., type and abundance of microbes) [118]. The bacterial DNA sequence found in healthy blood belongs mainly to the Firmicutes, Bacteroidetes, Proteobacteria, and Actinobacteria phyla [119].

This microbial ecosystem, co-evolved with humans across the millennia, is for the most part interactively co-dependent, both on one another and on their host, and capable of contributing and reacting to circulating signaling molecules [120]. Microbes in the gut produce a wealth of low-molecular-weight metabolites (metabolome), including trimethylamine N-oxide (TMAO), short-chain fatty acids (SCFAs), secondary bile acid, and indoxyl sulfate, from exogenous dietary substances or endogenous metabolic compounds. These microbial-derived metabolites are the major factors in the host–microbiota cross-talk by activation of numerous complex signaling pathways [121], such as the nuclear factor kappa-light-chain-enhancer of activated B cells, Bcl-2 interacting protein 3, NLR family pyrin domain containing inflammasome, and protein kinase RNA-like endoplasmic reticulum kinase [122], linked also to numerous types of programmed cell death, including apoptosis, autophagy, pyroptosis, ferroptosis, and clockophagy [123].

The view of humans as holobionts consisting of eukaryotic host cells and associated prokaryotic organisms has opened up a new perspective on cardiovascular pathophysiology leading to the Human Microbiome Project commencement [124]. The number of bacterial genes encoded within the human gut vastly outnumber the total complement of genes in *Homo sapiens*, endowing the gut microbiome with enormous potential for production of functionally active metabolites [125]. Microbiota-derived metabolites, such as SCFAs, primary and secondary bile acids (Bas), TMAO, lipopolysaccharides (LPS), uremic toxins, phenylacetylglutamine (PAGln), branched-chain aminoacids (BCAA), intestinal fatty-acid-binding protein (I-FABP), zonulin, and sphingomyelins, which are adsorbed in the intestine and distributed via the circulation, can exert beneficial or detrimental effects on various extraintestinal organs, including the brain, liver, and heart [23,126,127,128,129].

This virtually endocrine ‘organ’ plays an important role in the function of the gastrointestinal tract and in the human physiology, protecting from pathogen colonization through maintenance of the gut barrier function and participating in digestion, energy harvest from food sources indigestible by humans, vitamin synthesis, functions of the immune system, facilitation of biotransformation of drugs such as statins and antihypertensives, and regulation of brain function and behavior, as well as endocrine and glucose homeostasis and lipid and bile acid metabolism [96,121,130,131,132,133]. The importance of the gut–brain axis in regulating stress-related responses and anxiety disorders by influencing, in particular, tryptophan metabolism through the kynurenine pathway and consequently the serotoninergic system, has long been appreciated [134].

Many studies [91,112,116,117,119,129,135,136,137,138,139,140,141,142] have demonstrated that some microbial agents are positively and negatively associated with the presence of cardiovascular risk factors and various cardiovascular diseases, as well as with cardiovascular mortality; Table 2 summarizes the main results of these studies.

## 6. Western Diet, Microbiome, and Cardiovascular Diseases

The gut microbiota function as an endocrine organ that participates in the maintenance of cardiovascular homeostasis, and their dysfunction can directly influence the progression of cardiovascular disease [135] via abnormally activating signaling pathways, more swiftly when the gut barrier integrity is broken down (theory of “gut–heart axis”) [136].

Some microbial components are more represented in in the blood, fecal, and plaque samples of patients with cardiovascular risk factors or disease [91,116,117,119,137,138,139,140,141,142].

Host diet is one of the most significant modulators of the gut microbial community in humans, as well as in experimental animal models [96]. Bacterial metabolites are produced from food components, which in turn emphasizes the importance of nutrition. Evidence demonstrates that dietary habits such as the ‘Western diet’ model are related to perturbations in gut microbiome composition and function (called dysbiosis), with a significant decrease in Bacteroidetes and an increase in Firmicutes, *Escherichia*, *Shigella*, and *Enterococcus*, strongly associated with a wide range of human diseases, including celiac disease, inflammatory bowel disease, colorectal cancer, depression, anxiety, neurological disorders, rheumatoid arthritis, systemic lupus erythematous, asthma, allergies, insulin resistance, non-alcoholic fatty liver disease, chronic kidney disease, obesity, metabolic syndrome, arterial hypertension, type-2 diabetes and CVD [121,134,143,144,145,146,147,148,149,150,151,152,153]. A Western diet can lead to increased permeability of the gut mucosa, known as “leaky gut”, resulting in endotoxemia and bacterial translocation [12]. In turn, gut dysbiosis and impaired intestinal permeability can alter the gut bacterial metabolite signaling profile from the gut to the brain and heart [126,134].

Apart from diet, other conditions are associated with dysbiosis, e.g., antibiotics abuse [144]. In older age groups, there is an increase in microorganisms secreting endotoxins, LPS, and TMAO. Also, several pathological conditions in the gastrointestinal tract may impair the intestinal barrier, allowing translocation of bacteria and their metabolites [14].

The mechanisms linking gut microbiota to CVD are multifaceted and not yet fully understood and may include direct effects of microbial metabolites on atherosclerosis and thrombosis development, as well as immune dysregulation and disturbance of neuro-enteroendocrine hormones by bacteria and their products [125,144,147]. Borton et al. assigned an atherosclerotic profile to the 6341 microbial genomes that encoded metabolisms associated with heart disease, creating the open-access resource, the Methylated Amine Gene Inventory of Catabolism database (MAGICdb) [154].

One of the most-cited examples of the gut-microbiome-modulating human disease is the microbial metabolism of quaternary amines from protein-rich foods. Some species of the microbiota influence the metabolism of specific food components abundant in high-fat diets (such as carnitine, choline, betaine, and phosphatidyl-choline), synthesizing through lyase enzymes (catalytic protein cutC), trimethylamine (TMA) (humans lack this ability), which enters the liver through the portal vein circulation and is oxidized by the hepatic flavin-containing mono-oxygenase family to TMAO, a molecule with documented harmful activity on atherosclerosis and thrombosis in vitro and in vivo (it damages vascular endothelium and promotes activation of macrophages and platelets and thrombus formation) [96,124,126,132,155,156,157,158,159,160,161]. There was no direct association of plasma TMAO and the extent of atherosclerosis, both in mice and humans. However, TMAO plasma levels are associated with atherosclerotic plaque instability [162]. Associations with diabetes mellitus and obesity suggest that TMAO might have a functional role in metabolic syndrome [163]. In a community-based cohort of older US adults, after multivariable adjustment, higher levels of TMAO were associated with a higher risk of incident atherosclerotic cardiovascular disease (ASCVD) (HR 1.21 (95% CI: 1.02–1.42; *p*-trend = 0.029)) [164]. In the PEGASUS-TIMI 54 trial, higher TMAO quartiles were associated with risk of major adverse cardiovascular events (MACE) (OR 1.43, 95% CI: 1.06–1.93, *p* = 0.015) [165]. In patients with chronic heart failure after myocardial infarction, TMAO was a significant, independent predictor of MACE (HR 2.31, 95% CI: 1.42–3.59, *p* < 0.01) and all-cause mortality (HR 2.15, 95% CI: 1.37–3.24, *p* < 0.01) [166]. In a meta-analysis, a higher plasma TMAO level was associated with greater risks of MACEs (TMAO tertile 3 vs. tertile 1: HR, 1.68; 95% CI: 1.44–1.96) and of all-cause mortality (TMAO tertile 3 vs. tertile 1: HR, 1.67; 95% CI: 1.17–2.38) [167]. In another meta-analysis, high TMAO was positively associated with all-cause mortality (HR 1.38, 95% CI: 1.306–1.460), as well as adverse cardiovascular events (HR: 1.032, 95% CI: 1.014–1.051) [168].

There are obviously many other candidate mechanisms. Immune cells such as T cells, B cells, and macrophages are extensively infiltrated in the gut and heart tissues and play a crucial role in the crosstalk between the heart and gut microbiota [127,169]. Studies of germ-free mice have provided evidence that microbiota diversity and the presence of a specific microbe in the gut can affect immune cells in hosts [170]. Lower cholesterol-degrading bacteria were considerably reduced in myocardial infarct ion [119]. Dysbiosis with decreased abundance of microbes with capacity for producing butyrate, like chronic stress, decreases SCFAs and bile acids, raising intestinal permeability [118,157,171]. In diets high in saturated fat and low in fiber, enhanced absorption of bacterial fragments and bacterial fermentation end products, such as LPS, promotes the onset of “metabolic endotoxemia,” defined as a two- to threefold increase in circulating levels of bacterial endotoxin, which could activate toll-like receptors, mediating a chronic, low grade inflammatory response [119,153,172]. In atrial fibrillation, gut-derived LPS may contribute to MACE incidence by increasing platelet activation [173]. Additionally, gut microbiota may influence drug- and food-derived bioactive compounds metabolism. In periodontal disease, the oral microbiota is translocated through the bloodstream to the liver and intestine, generating intestinal dysbiosis [174]. Transfer of microbiota from obese animals induces metabolic disease and obesity in germ-free animals. Conversely, transfer of pathogen-free microbiota from lean healthy human donors to patients with metabolic disease can increase insulin sensitivity [150].

**Figure 1:**
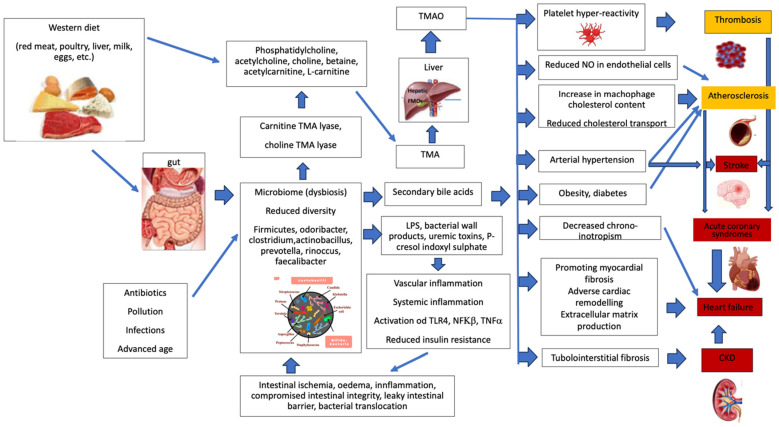
A tentative, synthetic description of the pathways related to Western diet, microbiome dysbiosis, and cardiovascular diseases. Abbreviations: CKD: chronic kidney disease; FMO3: flavin monooxygenase-3; LPS: lipopolysaccharide; NFκβ: nuclear factor κβ; NO: nitric oxide; TLR4: toll-like receptor-4; TMA: trimethylamine; TMAO: trimethylamine-N-oxide; TNFα: tumor necrosis factor α.

In heart failure (HF), current evidence has found links with alterations in microbial composition and function associated with impaired intestinal barrier function, generation of uremic toxins, and bacterial translocation leading to inflammatory and immune responses [175,176]. Intestinal leakage, caused by hemodynamic changes in heart failure (congestion in the portal vein, drop in cardiac output, and reduction in intestinal perfusion) induce, in turn, an alteration in gut microbiota composition and systemic inflammation through microbial or endotoxin translocation into systemic circulation (“gut hypothesis” of HF) [115,132,157,161,177]. Circulating TMAO levels are associated with adverse outcomes in HF [124,178]. Another effect of alteration in microbiota composition is reflected in the up-regulation of cotransporters (NHE3) with consequent salt and fluid overload [179]. Some hypothesized pathways connecting Western diet, microbiome, and CVD are summarized in Figure 1.

## 7. Effects of Mediterranean Diet on Microbiome

Although RCTs and observational studies provided no clear evidence of a consistent effect of an MD on the composition or metabolism of the gut microbiota [180], the consumption of a Mediterranean-type diet is associated with a specific microbiota characterized by a greater biodiversity (i.e., by a greater number of bacterial species identified and an increase in gene richness) and by fiber-degrading and butyrate-producing bacteria [13,90,91,96,105,114,148,155,181,182,183,184,185,186,187]. Table 3 shows the effects of MD on gut microbiota [90,91,96,105,114,148,155,181,187].

In summary, MD adherence is associated to an increase in the abundance of several Bacteroidetes taxa and a depletion of many Firmicutes taxa, with a lower Firmicutes/Bacteroidetes ratio and a higher bifidobacterial/*E. coli* ratio and *Prevotella*/*Bacteroides* ratio [183,185,188]. Cereal consumption is associated, for example, with the presence of *Bifidobacterium* and *Faecalibacterium*, olive oil consumption with *Tenericutes* and *Dorea*, red wine consumption with *Faecalibacterium*, vegetable consumption with *Rikenellaceae*, *Dorea*, *Alistipes*, and *Ruminococcus*, and legume consumption with *Coprococcus* [114].

Another interesting point is the food matrix effect of the Mediterranean diet and its actions on microbiota. The concept of a “food matrix”, in a simple manner, states that the different compounds located in the food, rather than the single nutrients, interact in a coordinated way in the human body, determining the benefits or dangers derived from food consumption [189]. In other words, the “food matrix” is a physical form constituting a certain food in which specific nutrients provide functionalities that are different from those exhibited by the same compounds when considered in isolation or a free state. The poor matrix of the Western-like diet generates an unfavorable environment in the gut and the microbiome, therefore leading to dysbiosis; in contrast, the Mediterranean diet, rich in plant-based aliments, presents a complex of elements that, in an adequate and complete food matrix, determines their beneficial properties in maintaining gut microbiota eubiosis [189].

## 8. MD, Microbiome, and Cardiovascular Health

The relationship between the gut microbiome, diet, and cardiovascular diseases is complex and still not fully understood. The microbiome could represent, however, a possible intermediate of the effects of the MD on modulation of cardiovascular risk factors [96]. An interesting hypothesis suggests a bidirectional relationship between the MD and the gut microbiome, where gut microbiota assembly and biosynthetic capacity are responsive to the diet; in return, the microbiome-reachable nutrients shape and modulate the microbiome toward a characteristic probiotic state. It can be speculated that the primary health benefits of the MD are mediated by the bioactive compounds transformed by the microbiome [190].

First, adherence to an MD led to a higher abundance of different taxa that are negatively correlated with markers of inflammation. This diet pattern positively affects the diversity and activity of various gut bacteria with saccharolytic activity (e.g., *Bacteroides acidifaciens*, *Firmicutes*, *Faecalibacterium prausnitzii*, *Prevotella*, *clostridium cluster XIVa*, *Akkermansia*, *Roseburia* and *Ruminococcus genera*, and *Parabacteroides distasonis*) that increases the SCFAs produced during microbial fermentation of complex carbohydrates and dietary fiber, hence improving host metabolism [13,91,96,148,155,185,191,192]. SCFAs, especially butyric acid but also acetate and propionate, possess immunomodulatory and anti-inflammatory properties (reducing some cytokines such as VEGF, MCP-1, IL-17, IP-10, and IL-12) and improve host metabolism [96,125,129,182,184,193]. Receptors binding these metabolites, such as G-protein-coupled receptors GPR41,GPR43, GPR109a, and OLF78, placed on enteroendocrine and immune cells have been shown in animal studies to have inverse roles in blood pressure regulation and favorably impact cardiac function [96,194].

Apart from SCFAs, the MD has some other interesting cardioprotective properties. The MD, and in particular fruit and legumes, is inversely related to LPS levels, linked with baseline urinary excretion of TxB2 [173]. An MD increases urinary urolithins, fecal bile acid degradation, and insulin sensitivity [91]. The MD is rich in polyphenols that are extensively metabolized by the gut microbiota. Among the five microbial phenolic metabolites identified, urolithin B glucuronide was inversely associated with LDL-cholesterol [195]. Some genera related to the MD seem to affect the bile acid metabolism. Bile acids represent a class of cholesterol derivatives that is essential for intestinal absorption of lipids and fat-soluble vitamins, playing an important modulator role in cholesterol turnover, in improvement in insulin levels, and in the control of immunity and heart function [90,196]. Some strains, on the other hand, produce secondary metabolites originating from molecules present in food (such as enterodiol, which derives from lignin), characterized by a vascular protection activity [155]. Finally, the Mediterranean diet pattern, rich in unsaturated fats and fiber, may be one dietary strategy to reduce metabolic endotoxemia-microbiome derived [197].

Preclinical studies have demonstrated the differential effects of MD on the microbiota and metabolic health [197]. A systematic review of animal studies shows that MD-like diets rich in polyphenol fiber modified the gut microbiota composition and increased microbial metabolites’ activities, leading to an improvement in HF outcomes, such as a reduction in systolic blood pressure, cardiac hypertrophy, and left ventricular thickness [160].

However, human studies are lacking. MD adherence results in a better glycemic control in subjects with T2D. Bacterial richness was negatively correlated with fasting glucose levels and the homeostatic model assessment for insulin resistance (HOMA-IR). Fecal alkaline phosphatase activity, positively correlated with bacterial diversity, was negatively correlated with HbA1c [188].

**Figure 2:**
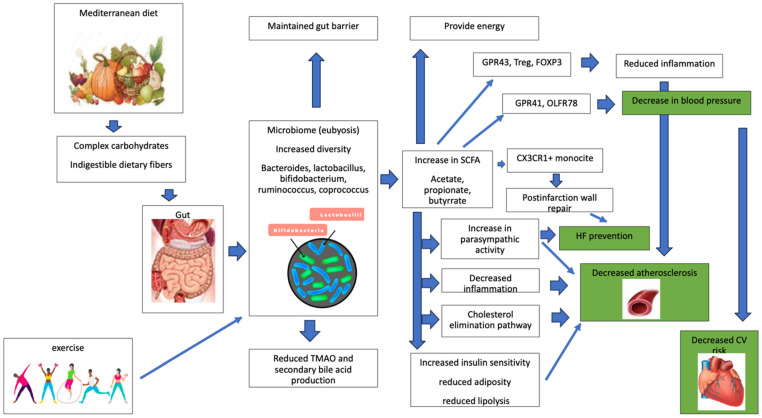
A tentative, synthetic description of the pathways related to the Mediterranean diet, microbiome eubiosis, and cardiovascular health. Abbreviations: CV: cardiovascular; CX3CR1: C-X3-C motif chemokine receptor 1; FOXP3 forkhead box P3: GPR: G protein receptor; HF: heart failure; OLFR: olfactory G-protein-coupled receptor-78: SCFA: short-chain fatty acids; TMAO: trimethylamine-N-oxide; Treg: regulatory T cells.

Some hypothesized pathways connecting MD, microbiome, and cardiovascular health are summarized in Figure 2.

## 9. Conclusions

The integration of microbiome analysis within nutrition science research will be fundamental to ensuring our full understanding of the complex and synergistic effects that foods or dietary patterns can have on human health. Intestinal microbiota are rising as a new element in the physiopathology of cardiovascular diseases. A healthy microbiota includes a balanced representation of bacteria with health promotion functions (symbiotes). It is rational to speculate that a positive modulation of the gut microbiome diversity, composition, and function is one of the main factors intermediating the health effects of MD on the host.

Further research is needed to explore the specific mechanisms underlying the protective effects of this dietary pattern and to better understand the long-term effects of the MD on atherosclerosis and its associated risk factors in diverse populations, as well as the therapeutic potential of the gut–metabolite–heart axis as a novel target for the treatment of CVD. As a consequence, more high-quality prospective cohorts and randomized clinical trials are warranted.

Nevertheless, promoting the adoption of the MD could be an effective strategy for mitigating the burden of CVDs globally [74]. We hope this article will draw the attention of society and the medical community to emphasize promoting healthy eating and proper eating habits in children and adults.

## Figures and Tables

**Table 1:** MD and cardiovascular outcomes: main studies.

Author, Year	Study Setting	Findings
De Lorgeril et al., 1999 [69]	Secondary prevention, intervention	Three composite outcomes (COs) combining either cardiac death and nonfatal myocardial infarction (CO 1), or the preceding plus major secondary end points (unstable angina, stroke, heart failure, and pulmonary or peripheral embolism) (CO 2), or the preceding plus minor events requiring hospital admission (CO 3) were studied. In the Mediterranean diet group, CO 1 was reduced (14 events versus 44 in the prudent Western-type diet group, *p* = 0.0001), as were CO 2 (27 events versus 90, *p* = 0.0001) and CO 3 (95 events versus 180, *p* = 0. 0002).
Martinez-Gonzalez et al., 2002 [77]	Observational case-control	For each additional point in the a priori Mediterranean pattern (observed range: 9–38) the odds ratio (95% CI) was 0.92 (0.86–0.98). This estimate was 0.55 (0.42–0.73) when using the post hoc pattern (range: 0–8).
Panagiotakos et al., 2002 [54]	Observational case-control	MeDiet reduces the risk of developing acute coronary syndromes by 17% (odds ratio = 0.83, 95% CI 0.73–0.88, *p* < 0.01) in controlled hypertensive subjects, and by 20% (odds ratio = 0.80, 95% CI 0.71–0.89, *p* < 0.01) in normotensive subjects.
Pitsavos et al., 2002 [56]	Observational case-control	The combination of a MeDiet and statin medical therapy is associated with an additional reduction in the coronary risk (odds ratio = 0.57, *p* < 0.01), independently from cholesterol levels and the other cardiovascular factors.
Panagiotakos et al., 2006 [55]	Observational	In acute coronary syndrome, an increment in the diet score was associated with a significant decrease in troponin I and creatine phosphokinase-MB levels (*p* < 0.01) after adjusting for various potential confounders. Moreover, diet score was associated with lower risk of recurrent events (odds ratio = 0.81, 95% CI 0.61–0.98).
Fung et al., 2009 [65]	Observational	Women in the top aMed quintile were at lower risk for both CHD and stroke compared with the bottom quintile (RR = 0.71 (95% CI = 0.62–0.82; *p* trend < 0.0001) for CHD; RR = 0.87 (95% CI = 0.73–1.02; *p* trend = 0.03) for stroke). CVD mortality was significantly lower among women in the top quintile of the aMed (RR = 0.61, 95% CI = 0.49–0.76, *p* trend < 0.0001).
Levitan et al., 2013 [61]	Observational, longitudinal	Multivariable-adjusted HRs were 1 (reference), 1.05 (95% CI 0.89–1.24), 0.97 (95% CI 0.81–1.17), and 0.85 (95% CI 0.70–1.02) across quartiles of the Mediterranean diet score (*p*-trend = 0.08)
Tektonidis et al., 2015 [57]	Observational, longitudinal	A high adherence to the mMED score (6–8), compared to low, was associated with a lower risk of MI (RR: 0.74, 95% CI: 0.61–0.90, *p* = 0.003), HF (RR: 0.79, 95% CI: 0.68–0.93, *p* = 0.004) and ischemic stroke (RR: 0.78, 95% CI: 0.65–0.93, *p* = 0.007) but not hemorrhagic stroke (RR: 0.88, 95% CI: 0.61–1.29, *p* = 0.53).
Tong et al., 2016 [67]	Observational	The Mediterranean diet score (MDS) was significantly associated with lower incidence of the cardiovascular outcomes, with hazard ratios (95% confidence intervals) of 0.95 (0.92–0.97) per one standard deviation for incident CVD and 0.91 (0.87–0.96) for CVD mortality. Associations were similar for composite incident ischaemic heart disease and all-cause mortality.
Liyanage et al., 2016 [76]	Meta-analysis	Evidence of protection against major vascular events (RR 0.63, 95% confidence interval 0.53–0.75), coronary events (0.65, 0.50–0.85), stroke (0.65, 0.48–0.88), and heart failure (0.30, 0.17–0.56) but not for all-cause mortality (1.00, 0.86–1.15) or cardiovascular mortality (0.90, 0.72–1.11).
Grosso et al., 2017 [78]	Meta-analysis	Individuals in the highest quantile of adherence to the diet had lower incidence [relative risk (RR): 0.76, 95% CI: 0.68, 0.83] and mortality (RR: 0.76, 95% CI: 0.68, 0.83) from CVD compared to those least adherent. A significant reduction in risk was found also for coronary heart disease (CHD) (RR: 0.72, 95% CI: 0.60, 0.86), myocardial infarction (MI) (RR: 0.67; 95% CI: 0.54, 0.83), and stroke (RR: 0.76; 95% CI: 0.60, 0.96) incidence.
Stefler et al., 2017 [68]	Observational	One standard deviation (SD) increase in the MDS (equivalent to a 2.2-point increase in the score) was found to be inversely associated with death from all causes (HR, 95% CI 0.93, 0.88–0.98) and CVD (0.90, 0.81–0.99) even after multivariable adjustment. An inverse but statistically not significant link was found for CHD (0.90, 0.78–1.03) and stroke (0.87, 0.71–1.07).
Bonaccio et al., 2017 [53]	Observational	A retrospective analysis of 18,991 men and women aged ≥35 years from the general population of the Moli-sani cohort (Italy). Overall, a two-point increase in MDS was associated with a 15% reduced CVD risk (95% confidence interval: 1% to 27%). Such association was evident in highly (HR = 0.43; 0.25–0.72) but not in less (HR = 0.94; 0.78–1.14) educated subjects (*p* for interaction = 0.042). Similarly, CVD advantages associated with the MD were confined to the high household income group (HR = 0.39; 0.23–0.66 and HR = 1.01; 0.79–1.29 for high- and low-income groups, respectively); *p* for interaction = 0.0098.
Dinu et al., 2018 [81]	Umbrella review of 13 meta-analyses of observational studies and 16 meta-analyses of RCTs	Robust evidence, supported by a *p*-value < 0.001, a large simple size, and a not considerable heterogeneity between studies, for a greater adherence to the Mediterranean diet and a reduced risk of overall mortality, cardiovascular diseases, coronary heart disease, myocardial infarction, overall cancer incidence, neurodegenerative diseases, and diabetes was found.
Waldeyer et al., 2018 [60]	Observational study	In patients undergoing coronary angiography, adherence to MD represented by a higher MDS was significantly associated with a reduced probability for a medium/high risk SYNTAX score of ≥23 with an odds ratio (OR) of 0.923 per point increase in MDS (95% confidence interval 0.869–0.979; *p* = 0.0079).
Shikany et al., 2018 [62]	Observational, longitudinal	In multivariable-adjusted models, the Mediterranean diet score was inversely associated with the hazard of recurrent CHD events (hazard ratio for highest score versus lowest score, 0.78; 95% confidence interval, 0.62–0.98; PTrend = 0.036).
Hodge et al., 2018 [63]	Observational, longitudinal	The hazard ratio for the total was 0.86 (95% CI: 0.80–0.93) comparing the highest and lowest three categories of MDS.
Mirò et al., 2018 [59]	Observational, longitudinal	In patients diagnosed with AHF after a mean follow-up period of 2 years, no differences were observed in survival between adherent and nonadherent patients (HR of adherents 0.86; 95% CI: 0.73 to 1.02). Adherence to the MD was associated with decreased rates of rehospitalization during the next year.
Rosato et al., 2019 [79]	Meta-analysis	The RR for the highest versus the lowest category of the MDS was 0.81 (95% CI: 0.74–0.88) for the 11 studies that considered unspecified CVD. The corresponding pooled RR for CHD/AMI risk was 0.70 (95% CI: 0.62–0.80). The overall RR for the six studies that considered unspecified stroke was 0.73 (95% CI: 0.59–0.91) for the highest versus the lowest category of the MDS. The corresponding values were 0.82 (95% CI: 0.73–0.92) for ischemic (five studies) and 1.01 (95% CI: 0.74–1.37) for hemorrhagic stroke.
Saulle et al., 2019 [75]	Systematic review	The Mediterranean diet may be a useful means of preventing stroke; the 6 meta-analyses especially highlighted that high adherence to the Mediterranean diet was protective against stroke, with a relative risk ranging from 0.64 (95% CI: 0.48–0.88) to 0.90 (95% CI: 0.87–0.93).
Delgado-Lista et al., 2022 [71]	Secondary prevention, intervention	Multivariable-adjusted hazard ratios (HRs) of the different models ranged from 0.719 (95% CI: 0.541–0.957) to 0.753 (0.568–0.998) in favor of the Mediterranean diet.
Chang et al., 2022 [64]	observational	The alternate Mediterranean diet index aMED 3 (vs. <3) was not associated with a lower risk of all-cause (adjusted HR 0.797, 95% CI: 0.599–1.059, *p* = 0.116) and cardiovascular (adjusted HR 0.911, 95% CI: 0.539–1.538, *p* = 0.724) mortality in participants with a history of heart failure.
Liang et al., 2022 [66]	Observational	In Cox regression analysis, a higher absolute aMED score (HR 0.798, *p* = 0.0079) or an above-median aMED score (score 4–9) (HR 0.646, *p* = 0.0013) was negatively associated with all-cause mortality. In contrast, a higher aMEDscore was not associated with less cardiovascular mortality.
Gupta et al., 2023 [73]	Systematic review	Consumption of an MD associated with longer life and lower incidence of heart disease.
Laffond et al., 2023 [74]	Systematic review	Higher adherence to the MD is associated with a reduced risk of overall mortality, both in the general population and in patients with previous CVDs. Moreover, evidence suggests that following this dietary pattern likely decreases the risk of CVDs such as heart attacks, various types of coronary artery disease, stroke, and cardiovascular mortality.
Cangemi et al., 2023 [52]	Observational	In multivariate Cox regression analysis, a greater adherence remained inversely associated with major adverse cardiovascular events (HR: 0.49; 95% CI: 0.29–0.82; *p* = 0.006) after adjusting for confounding factors.
Pant et al., 2023 [80]	Meta-analysis	In women, higher adherence to a Mediterranean diet was associated with a lower CVD incidence (HR 0.76, 95% CI: 0.72 to 0.81; I2 = 39%, *p* test for heterogeneity = 0.07), total mortality (HR 0.77, 95% CI: 0.74 to 0.80; I2 = 21%, *p* test for heterogeneity = 0.28), and coronary heart disease (HR 0.75, 95% CI: 0.65 to 0.87; I2 = 21%, *p* test for heterogeneity = 0.28). Stroke incidence was lower in women with higher Mediterranean diet adherence (HR 0.87, 95% CI: 0.76 to 1.01; I2 = 0%, *p* test for heterogeneity = 0.89), but this result was not statistically significant.

**Table 2:** Main components of microbiome in cardiovascular diseases (from [91,112,116,117,119,129,135,136,137,138,139,140,141,142]).

Disease/Condition	Main Microbial Agents
Cardiovascular risk factors	*Prevotella 2*, *Prevotella 7*, *Tyzzerella* and *Tyzzerella 4* genera, *Bacteroides uniformis* and *B. vulgatus* (low prevalence of *Alloprevotella Prevotella copri* and *Catenibacterium*)
Arterial hypertension	*Catabacter*, *Robinsoleilla*, *Serratia*, *Enterobacteriaceae*, *Ruminococcus torques*, *Parasutterella*, *Escherichia*, *Shigella*, and *Klebsiella* (decreased abundance of *Sporobacter*, *Roseburia hominis*, *Romboutsia* spp., and *Roseburia*)
Atrial fibrillation	Enorma and *Bifidobacterium* genera
Diabetes mellitus	Order *Rhizobiales*, family *Desulfovibrionaceae*, genus *Romboutsia*
Coronary heart disease	*Proteobacteria* and *Actinobacteria phyla*, *Bacteroides*, *Prevotella*, *Firmicutes*, *Veillonella*, *Clostridium*, *Lactobacillaceae* (*Lactobacillus plantarum*) and *Streptococcus* (decreased prevalence of *Caulobacterales* order and *Caulobacteraceae* family, *aminococcaceae* and *Odoribacteraceae*)
Cerebrovascular disease	*Firmicutes*, *Proteobacteria*, and *Actinobacteria phyla*
Heart failure	*Ruminococcus gnavus*, *Escherichia Shigella*, *Streptococcus* sp. (*sanguinus* and *parasanguinis*), *Veillonella* sp., and *Actinobacteria* (relative depletion of *Eubacterium*, *Prevotella*, *Faecalibacterium*, *SMB53*, *aminococcaceae*, *Odoribacteraceae* and *Megamonas*)
Cardiovascular mortality	Genera *Kocuria* and *Enhydrobacter* (genera *Paracoccus* was inversely related)

**Table 3:** Effects of MD on gut microbiota (from [90,91,96,105,114,148,155,181,187]).

Increase	Decrease
*Akkermansia muciniphila*, *Anaerostipes hadrus*, *Bacteroides thetaiotaomicron*, *Bifidobacteria animalis*, *Candida albicans*, *Catenibacterium*, *Christensenellaceae*, *Clostridium* (*cluster XIVa*, *leptum*) *Enterorhabdus*, *Eubacterium rectale*, *Faecalibacterium* (*Lactococcus*, *prausnitzii*) *Lachnoclostridium*, *Lachnospiraceae*, *Oscillospira* (*Flavonifractor*), *Parabacteroides*, *Phascolarctobacterium*, *Prevotellaceae*, *Prevotellae*, *Proteobacteria*, *Roseburia faecis*, *Ruminococcaceae bromii* and *plautii*, *Sphingobacteriaceae*	*Actinomyces lignae*, *Butyricicoccus*, *Catenibacterium*, *Clostridium ramosum*, *Collinsella aerofaciens*, *Coprococcus Anaerostipes and comes*, *Dorea formicigenerans*, *Escherichia coli*, *Eubacterium hallii*, *Firmicutes*, *Flavonifractor plautii*, *Haemophilus*, *Lachnospiraceae* *Megamonas*, *Ruminiclostridium*, *Ruminococcus gnavus* and *torques* *Veillonella dispar*

## References

[1] Martínez-González M.A., Gea A., Ruiz-Canela M. (2019). The Mediterranean Diet and Cardiovascular Health: A Critical Review. Circ. Res..

[2] Hernando-Requejo O., García De Quinto H. (2021). Mediterranean Diet and Cancer. Nutr. Hosp..

[3] Dussaillant C., Echeverría G., Urquiaga I., Velasco N., Rigotti A. (2016). Evidencia actual sobre los beneficios de la dieta mediterránea en salud. Rev. Méd. Chile.

[4] Urquiaga I., Echeverría G., Dussaillant C., Rigotti A. (2017). Origen, componentes y posibles mecanismos de acción de la dieta mediterránea. Rev. Méd. Chile.

[5] Urpi-Sarda M., Casas R., Chiva-Blanch G., Romero-Mamani E.S., Valderas-Martínez P., Arranz S., Andres-Lacueva C., Llorach R., Medina-Remón A., Lamuela-Raventos R.M. (2012). Virgin olive oil and nuts as key foods of the Mediterranean diet effects on inflammatory biomarkers related to atherosclerosis. Pharmacol. Res..

[6] Davis C., Bryan J., Hodgson J., Murphy K. (2015). Definition of the Mediterranean Diet; A Literature Review. Nutrients.

[7] Bach-Faig A., Berry E.M., Lairon D., Reguant J., Trichopoulou A., Dernini S., Medina F.X., Battino M., Belahsen R., Miranda G. (2011). Mediterranean diet pyramid today. Science and cultural updates. Public Health Nutr..

[8] Kahleova H., Levin S., Barnard N.D. (2018). Vegetarian Dietary Patterns and Cardiovascular Disease. Prog. Cardiovasc. Dis..

[9] Sofi F., Dinu M., Pagliai G., Cesari F., Marcucci R., Casini A. (2016). Mediterranean versus vegetarian diet for cardiovascular disease prevention (the CARDIVEG study): Study protocol for a randomized controlled trial. Trials.

[10] O’Neill B., Raggi P. (2020). The ketogenic diet: Pros and cons. Atherosclerosis.

[11] Christ A., Lauterbach M., Latz E. (2019). Western Diet and the Immune System: An Inflammatory Connection. Immunity.

[12] Merra G., Noce A., Marrone G., Cintoni M., Tarsitano M.G., Capacci A., De Lorenzo A. (2020). Influence of Mediterranean Diet on Human Gut Microbiota. Nutrients.

[13] Muralidharan J., Moreno-Indias I., Bulló M., Lopez J.V., Corella D., Castañer O., Vidal J., Atzeni A., Fernandez-García J.C., Torres-Collado L. (2021). Effect on gut microbiota of a 1-y lifestyle intervention with Mediterranean diet compared with energy-reduced Mediterranean diet and physical activity promotion: PREDIMED-Plus Study. Am. J. Clin. Nutr..

[14] Ghosh T.S., Rampelli S., Jeffery I.B., Santoro A., Neto M., Capri M., Giampieri E., Jennings A., Candela M., Turroni S. (2020). Mediterranean diet intervention alters the gut microbiome in older people reducing frailty and improving health status: The NU-AGE 1-year dietary intervention across five European countries. Gut.

[15] Meng S., Cui Z., Li M., Li T., Wu F., Kang T., Meng H. (2021). Associations between Dietary Animal and Plant Protein Intake and Cardiometabolic Risk Factors—A Cross-Sectional Study in China Health and Nutrition Survey. Nutrients.

[16] Cani P.D., Van Hul M. (2020). Mediterranean diet, gut microbiota and health: When age and calories do not add up!. Gut.

[17] Kiani A.K., Medori M.C., Bonetti G., Aquilanti B., Velluti V., Matera G., Iaconelli A., Stuppia L., Connelly S.T., Herbst K.L. (2022). Modern vision of the Mediterranean diet. J. Prev. Med. Hyg..

[18] Bucciantini M., Leri M., Nardiello P., Casamenti F., Stefani M. (2021). Olive Polyphenols: Antioxidant and Anti-Inflammatory Properties. Antioxidants.

[19] Casamenti F., Stefani M. (2017). Olive polyphenols: New promising agents to combat aging-associated neurodegeneration. Expert Rev. Neurother..

[20] Barnes S., Prasain J., D’Alessandro T., Arabshahi A., Botting N., Lila M.A., Jackson G., Janle E.M., Weaver C.M. (2011). The metabolism and analysis of isoflavones and other dietary polyphenols in foods and biological systems. Food Funct..

[21] Peng Y., Hou C., Yang Z., Li C., Jia L., Liu J., Tang Y., Shi L., Li Y., Long J. (2016). Hydroxytyrosol mildly improve cognitive function independent of APP processing in APP/PS1 mice. Mol. Nutr. Food Res..

[22] Soto-Alarcon S.A., Valenzuela R., Valenzuela A., Videla L.A. (2017). Liver Protective Effects of Extra Virgin Olive Oil: Interaction between Its Chemical Composition and the Cell-signaling Pathways Involved in Protection. Endocr. Metab. Immune Disord.-Drug Targets.

[23] Ahmadmehrabi S., Tang W.H.W. (2017). Gut microbiome and its role in cardiovascular diseases. Curr. Opin. Cardiol..

[24] Hornedo-Ortega R., Cerezo A.B., De Pablos R.M., Krisa S., Richard T., García-Parrilla M.C., Troncoso A.M. (2018). Phenolic Compounds Characteristic of the Mediterranean Diet in Mitigating Microglia-Mediated Neuroinflammation. Front. Cell. Neurosci..

[25] Naureen Z., Capodicasa N., Paolacci S., Anpilogov K., Dautaj A., Dhuli K., Camilleri G., Connelly S.T., Gasparetto A., Bertelli M. (2021). Prevention of the proliferation of oral pathogens due to prolonged mask use based on α-cyclodextrin and hydroxytyrosol mouthwash. Eur. Rev. Med. Pharmacol. Sci..

[26] Downer M.K., Gea A., Stampfer M., Sánchez-Tainta A., Corella D., Salas-Salvadó J., Ros E., Estruch R., Fitó M., Gómez-Gracia E. (2016). Predictors of short- and long-term adherence with a Mediterranean-type diet intervention: The PREDIMED randomized trial. Int. J. Behav. Nutr. Phys. Act..

[27] Ros E. (2015). Nuts and CVD. Br. J. Nutr..

[28] Williamson G. (2017). The role of polyphenols in modern nutrition. Nutr. Bull..

[29] Loke W.M., Hodgson J.M., Proudfoot J.M., McKinley A.J., Puddey I.B., Croft K.D. (2008). Pure dietary flavonoids quercetin and (−)-epicatechin augment nitric oxide products and reduce endothelin-1 acutely in healthy men. Am. J. Clin. Nutr..

[30] Eisenberg T., Abdellatif M., Schroeder S., Primessnig U., Stekovic S., Pendl T., Harger A., Schipke J., Zimmermann A., Schmidt A. (2016). Cardioprotection and lifespan extension by the natural polyamine spermidine. Nat. Med..

[31] Buil-Cosiales P., Toledo E., Salas-Salvadó J., Zazpe I., Farràs M., Basterra-Gortari F.J., Diez-Espino J., Estruch R., Corella D., Ros E. (2016). Association between dietary fibre intake and fruit, vegetable or whole-grain consumption and the risk of CVD: Results from the PREvención con DIeta MEDiterránea (PREDIMED) trial. Br. J. Nutr..

[32] Pereira A., Maraschin M. (2015). Banana (*Musa* spp) from peel to pulp: Ethnopharmacology, source of bioactive compounds and its relevance for human health. J. Ethnopharmacol..

[33] Borgonovi T.F., Virgolin L.B., Janzantti N.S., Casarotti S.N., Penna A.L.B. (2022). Fruit bioactive compounds: Effect on lactic acid bacteria and on intestinal microbiota. Food Res. Int..

[34] Thorburn A.N., Macia L., Mackay C.R. (2014). Diet, Metabolites, and “Western-Lifestyle” Inflammatory Diseases. Immunity.

[35] Clinton S.K., Giovannucci E.L., Hursting S.D. (2020). The World Cancer Research Fund/American Institute for Cancer Research Third Expert Report on Diet, Nutrition, Physical Activity, and Cancer: Impact and Future Directions. J. Nutr..

[36] Surh Y.-J. (2003). Cancer chemoprevention with dietary phytochemicals. Nat. Rev. Cancer.

[37] Salque M., Bogucki P.I., Pyzel J., Sobkowiak-Tabaka I., Grygiel R., Szmyt M., Evershed R.P. (2013). Earliest evidence for cheese making in the sixth millennium bc in northern Europe. Nature.

[38] Şanlier N., Gökcen B.B., Sezgin A.C. (2019). Health benefits of fermented foods. Crit. Rev. Food Sci. Nutr..

[39] Barengolts E., Smith E., Reutrakul S., Tonucci L., Anothaisintawee T. (2019). The Effect of Probiotic Yogurt on Glycemic Control in Type 2 Diabetes or Obesity: A Meta-Analysis of Nine Randomized Controlled Trials. Nutrients.

[40] Oliveira D.L., Costabile A., Wilbey R.A., Grandison A.S., Duarte L.C., Roseiro L.B. (2012). In vitro evaluation of the fermentation properties and potential prebiotic activity of caprine cheese whey oligosaccharides in batch culture systems. BioFactors.

[41] Cani P., Delzenne N. (2009). The Role of the Gut Microbiota in Energy Metabolism and Metabolic Disease. Curr. Pharm. Des..

[42] Jiang L., Wang J., Xiong K., Xu L., Zhang B., Ma A. (2021). Intake of Fish and Marine n-3 Polyunsaturated Fatty Acids and Risk of Cardiovascular Disease Mortality: A Meta-Analysis of Prospective Cohort Studies. Nutrients.

[43] Siscovick D.S., Barringer T.A., Fretts A.M., Wu J.H.Y., Lichtenstein A.H., Costello R.B., Kris-Etherton P.M., Jacobson T.A., Engler M.B., Alger H.M. (2017). Omega-3 Polyunsaturated Fatty Acid (Fish Oil) Supplementation and the Prevention of Clinical Cardiovascular Disease: A Science Advisory from the American Heart Association. Circulation.

[44] Teng M., Zhao X., Wang C., Wang C., White J.C., Zhao W., Zhou L., Duan M., Wu F. (2022). Polystyrene Nanoplastics Toxicity to Zebrafish: Dysregulation of the Brain–Intestine–Microbiota Axis. ACS Nano.

[45] Wu H., Guo J., Yao Y., Xu S. (2022). Polystyrene nanoplastics induced cardiomyocyte apoptosis and myocardial inflammation in carp by promoting ROS production. Fish Shellfish Immunol..

[46] Vázquez-Fresno R., Llorach R., Perera A., Mandal R., Feliz M., Tinahones F.J., Wishart D.S., Andres-Lacueva C. (2016). Clinical phenotype clustering in cardiovascular risk patients for the identification of responsive metabotypes after red wine polyphenol intake. J. Nutr. Biochem..

[47] Morselli E., Mariño G., Bennetzen M.V., Eisenberg T., Megalou E., Schroeder S., Cabrera S., Bénit P., Rustin P., Criollo A. (2011). Spermidine and resveratrol induce autophagy by distinct pathways converging on the acetylproteome. J. Cell Biol..

[48] Corella D., Coltell O., Macian F., Ordovás J.M. (2018). Advances in Understanding the Molecular Basis of the Mediterranean Diet Effect. Annu. Rev. Food Sci. Technol..

[49] De Meyer G.R.Y., Grootaert M.O.J., Michiels C.F., Kurdi A., Schrijvers D.M., Martinet W. (2015). Autophagy in Vascular Disease. Circ. Res..

[50] Bravo-San Pedro J.M., Kroemer G., Galluzzi L. (2017). Autophagy and Mitophagy in Cardiovascular Disease. Circ. Res..

[51] Razani B., Feng C., Coleman T., Emanuel R., Wen H., Hwang S., Ting J.P., Virgin H.W., Kastan M.B., Semenkovich C.F. (2012). Autophagy Links Inflammasomes to Atherosclerotic Progression. Cell Metab..

[52] Cangemi R., Miglionico M., D’Amico T., Fasano S., Proietti M., Romiti G.F., Corica B., Stefanini L., Tanzilli G., Basili S. (2023). Adherence to the Mediterranean Diet in Preventing Major Cardiovascular Events in Patients with Ischemic Heart Disease: The EVA Study. Nutrients.

[53] Bonaccio M., Di Castelnuovo A., Pounis G., Costanzo S., Persichillo M., Cerletti C., Donati M.B., De Gaetano G., Iacoviello L., on behalf of the Moli-sani Study Investigators (2017). High adherence to the Mediterranean diet is associated with cardiovascular protection in higher but not in lower socioeconomic groups: Prospective findings from the Moli-sani study. Int. J. Epidemiol..

[54] Panagiotakos D.B., Chrysohoou C., Pitsavos C., Tzioumis K., Papaioannou I., Stefanadis C., Toutouzas P. (2002). The association of Mediterranean diet with lower risk of acute coronary syndromes in hypertensive subjects. Int. J. Cardiol..

[55] Panagiotakos D.B., Arapi S., Pitsavos C., Antonoulas A., Mantas Y., Zombolos S., Stefanadis C. (2006). The relationship between adherence to the Mediterranean diet and the severity and short-term prognosis of acute coronary syndromes (ACS): The Greek Study of ACS (The GREECS). Nutrition.

[56] Pitsavos C., Panagiotakos D.B., Chrysohoou C., Skoumas J., Papaioannou I., Stefanadis C., Toutouzas P.K. (2002). The effect of Mediterranean diet on the risk of the development of acute coronary syndromes in hypercholesterolemic people: A case–control study (CARDIO2000). Coron. Artery Dis..

[57] Tektonidis T.G., Åkesson A., Gigante B., Wolk A., Larsson S.C. (2015). A Mediterranean diet and risk of myocardial infarction, heart failure and stroke: A population-based cohort study. Atherosclerosis.

[58] Martínez-González M.A., García-López M., Bes-Rastrollo M., Toledo E., Martínez-Lapiscina E.H., Delgado-Rodriguez M., Vazquez Z., Benito S., Beunza J.J. (2010). Mediterranean diet and the incidence of cardiovascular disease: A Spanish cohort. Nutr. Metab. Cardiovasc. Dis..

[59] Miró Ò., Estruch R., Martín-Sánchez F.J., Gil V., Jacob J., Herrero-Puente P., Herrera Mateo S., Aguirre A., Andueza J.A., Llorens P. (2018). Adherence to Mediterranean Diet and All-Cause Mortality After an Episode of Acute Heart Failure. JACC Heart Fail..

[60] Waldeyer C., Brunner F.J., Braetz J., Ruebsamen N., Zyriax B.-C., Blaum C., Kroeger F., Kohsiack R., Schrage B., Sinning C. (2018). Adherence to Mediterranean diet, high-sensitive C-reactive protein, and severity of coronary artery disease: Contemporary data from the INTERCATH cohort. Atherosclerosis.

[61] Levitan E.B., Lewis C.E., Tinker L.F., Eaton C.B., Ahmed A., Manson J.E., Snetselaar L.G., Martin L.W., Trevisan M., Howard B.V. (2013). Mediterranean and DASH Diet Scores and Mortality in Women with Heart Failure: The Women’s Health Initiative. Circ. Heart Fail..

[62] Shikany J.M., Safford M.M., Bryan J., Newby P.K., Richman J.S., Durant R.W., Brown T.M., Judd S.E. (2018). Dietary Patterns and Mediterranean Diet Score and Hazard of Recurrent Coronary Heart Disease Events and All-Cause Mortality in the REGARDS Study. J. Am. Heart Assoc..

[63] Hodge A.M., Bassett J.K., Dugué P.-A., Shivappa N., Hébert J.R., Milne R.L., English D.R., Giles G.G. (2018). Dietary inflammatory index or Mediterranean diet score as risk factors for total and cardiovascular mortality. Nutr. Metab. Cardiovasc. Dis..

[64] Chang C.-Y., Lee C.-L., Liu W.-J., Wang J.-S. (2022). Association of Adherence to the Mediterranean Diet with All-Cause Mortality in Subjects with Heart Failure. Nutrients.

[65] Fung T.T., Rexrode K.M., Mantzoros C.S., Manson J.E., Willett W.C., Hu F.B. (2009). Mediterranean Diet and Incidence of and Mortality from Coronary Heart Disease and Stroke in Women. Circulation.

[66] Liang K.-W., Lee C.-L., Liu W.-J. (2022). Lower All-Cause Mortality for Coronary Heart or Stroke Patients Who Adhere Better to Mediterranean Diet-An NHANES Analysis. Nutrients.

[67] Tong T.Y.N., Wareham N.J., Khaw K.-T., Imamura F., Forouhi N.G. (2016). Prospective association of the Mediterranean diet with cardiovascular disease incidence and mortality and its population impact in a non-Mediterranean population: The EPIC-Norfolk study. BMC Med..

[68] Stefler D., Malyutina S., Kubinova R., Pajak A., Peasey A., Pikhart H., Brunner E.J., Bobak M. (2017). Mediterranean diet score and total and cardiovascular mortality in Eastern Europe: The HAPIEE study. Eur. J. Nutr..

[69] De Lorgeril M., Salen P., Martin J.-L., Monjaud I., Delaye J., Mamelle N. (1999). Mediterranean Diet, Traditional Risk Factors, and the Rate of Cardiovascular Complications After Myocardial Infarction: Final Report of the Lyon Diet Heart Study. Circulation.

[70] Martínez-González M.A., Fernández-Jarne E., Serrano-Martínez M., Marti A., Martinez J.A., Martín-Moreno J.M. (2002). Mediterranean diet and reduction in the risk of a first acute myocardial infarction: An operational healthy dietary score. Eur. J. Nutr..

[71] Delgado-Lista J., Alcala-Diaz J.F., Torres-Peña J.D., Quintana-Navarro G.M., Fuentes F., Garcia-Rios A., Ortiz-Morales A.M., Gonzalez-Requero A.I., Perez-Caballero A.I., Yubero-Serrano E.M. (2022). Long-term secondary prevention of cardiovascular disease with a Mediterranean diet and a low-fat diet (CORDIOPREV): A randomised controlled trial. Lancet.

[72] Rees K., Takeda A., Martin N., Ellis L., Wijesekara D., Vepa A., Das A., Hartley L., Stranges S. (2019). Mediterranean-style diet for the primary and secondary prevention of cardiovascular disease. Cochrane Database Syst. Rev..

[73] Gupta U.C., Gupta S.C., Gupta S.S. (2023). An Evidence Base for Heart Disease Prevention using a MediterraneanDiet Comprised Primarily of Vegetarian Food. Recent Adv. Food Nutr. Agric..

[74] Laffond A., Rivera-Picón C., Rodríguez-Muñoz P.M., Juárez-Vela R., Ruiz De Viñaspre-Hernández R., Navas-Echazarreta N., Sánchez-González J.L. (2023). Mediterranean Diet for Primary and Secondary Prevention of Cardiovascular Disease and Mortality: An Updated Systematic Review. Nutrients.

[75] Saulle R., Lia L., De Giusti M. (2019). A systematic overview of the scientific literature on the association between Mediterranean Diet and the Stroke prevention. Clin. Ter..

[76] Liyanage T., Ninomiya T., Wang A., Neal B., Jun M., Wong M.G., Jardine M., Hillis G.S., Perkovic V. (2016). Effects of the Mediterranean Diet on Cardiovascular Outcomes—A Systematic Review and Meta-Analysis. PLoS ONE.

[77] Martinez-Gonzalez M.A., Bes-Rastrollo M. (2014). Dietary patterns, Mediterranean diet, and cardiovascular disease. Curr. Opin. Lipidol..

[78] Grosso G., Marventano S., Yang J., Micek A., Pajak A., Scalfi L., Galvano F., Kales S.N. (2017). A comprehensive meta-analysis on evidence of Mediterranean diet and cardiovascular disease: Are individual components equal?. Crit. Rev. Food Sci. Nutr..

[79] Rosato V., Temple N.J., La Vecchia C., Castellan G., Tavani A., Guercio V. (2019). Mediterranean diet and cardiovascular disease: A systematic review and meta-analysis of observational studies. Eur. J. Nutr..

[80] Pant A., Gribbin S., McIntyre D., Trivedi R., Marschner S., Laranjo L., Mamas M.A., Flood V., Chow C.K., Zaman S. (2023). Primary prevention of cardiovascular disease in women with a Mediterranean diet: Systematic review and meta-analysis. Heart.

[81] Dinu M., Pagliai G., Casini A., Sofi F. (2018). Mediterranean diet and multiple health outcomes: An umbrella review of meta-analyses of observational studies and randomised trials. Eur. J. Clin. Nutr..

[82] Abrignani M.G., Lucà F., Favilli S., Benvenuto M., Rao C.M., Di Fusco S.A., Gabrielli D., Gulizia M.M., On behalf of Cardiovascular Prevention Area, Young Cardiologists Area, and Paediatric Cardiology Task Force of the Associazione Nazionale Medici Cardiologi Ospedalieri (ANMCO), and Heart Care Foundation (2019). Lifestyles and Cardiovascular Prevention in Childhood and Adolescence. Pediatr. Cardiol..

[83] Capra M., Monopoli D., Decarolis N., Giudice A., Stanyevic B., Esposito S., Biasucci G. (2023). Dietary Models and Cardiovascular Risk Prevention in Pediatric Patients. Nutrients.

[84] Buckland G., Northstone K., Emmett P.M., Taylor C.M. (2024). Associations of childhood diet quality scores with arterial stiffness and carotid artery intima-media thickness in adolescence/early adulthood: Findings from the ALSPAC cohort. Br. J. Nutr..

[85] La Torre G., Saulle R., Di Murro F., Siliquini R., Firenze A., Maurici M., Mannocci A., Colamesta V., Barillà F., Ferrante F. (2018). Mediterranean diet adherence and synergy with acute myocardial infarction and its determinants: A multicenter case-control study in Italy. PLoS ONE.

[86] Korakas E., Dimitriadis G., Raptis A., Lambadiari V. (2018). Dietary Composition and Cardiovascular Risk: A Mediator or a Bystander?. Nutrients.

[87] Martínez-González M.Á., Ruiz-Canela M., Hruby A., Liang L., Trichopoulou A., Hu F.B. (2016). Intervention Trials with the Mediterranean Diet in Cardiovascular Prevention: Understanding Potential Mechanisms through Metabolomic Profiling. J. Nutr..

[88] Gepner Y., Shelef I., Komy O., Cohen N., Schwarzfuchs D., Bril N., Rein M., Serfaty D., Kenigsbuch S., Zelicha H. (2019). The beneficial effects of Mediterranean diet over low-fat diet may be mediated by decreasing hepatic fat content. J. Hepatol..

[89] Whayne T.F. (2014). Ischemic Heart Disease and the Mediterranean Diet. Curr. Cardiol. Rep..

[90] Galié S., García-Gavilán J., Papandreou C., Camacho-Barcía L., Arcelin P., Palau-Galindo A., Rabassa A., Bulló M. (2021). Effects of Mediterranean Diet on plasma metabolites and their relationship with insulin resistance and gut microbiota composition in a crossover randomized clinical trial. Clin. Nutr..

[91] Meslier V., Laiola M., Roager H.M., De Filippis F., Roume H., Quinquis B., Giacco R., Mennella I., Ferracane R., Pons N. (2020). Mediterranean diet intervention in overweight and obese subjects lowers plasma cholesterol and causes changes in the gut microbiome and metabolome independently of energy intake. Gut.

[92] Fitó M., Estruch R., Salas-Salvadó J., Martínez-Gonzalez M.A., Arós F., Vila J., Corella D., Díaz O., Sáez G., De La Torre R. (2014). Effect of the Mediterranean diet on heart failure biomarkers: A randomized sample from the PREDIMED trial. Eur. J. Heart Fail..

[93] Solá R., Fitó M., Estruch R., Salas-Salvadó J., Corella D., De La Torre R., Muñoz M.A., Del Carmen López-Sabater M., Martínez-González M.-A., Arós F. (2011). Effect of a traditional Mediterranean diet on apolipoproteins B, A-I, and their ratio: A randomized, controlled trial. Atherosclerosis.

[94] Casas R., Sacanella E., Urpí-Sardà M., Chiva-Blanch G., Ros E., Martínez-González M.-A., Covas M.-I., Lamuela-Raventos R.M., Salas-Salvadó J., Fiol M. (2014). The Effects of the Mediterranean Diet on Biomarkers of Vascular Wall Inflammation and Plaque Vulnerability in Subjects with High Risk for Cardiovascular Disease. A Randomized Trial. PLoS ONE.

[95] Giroli M.G., Werba J.P., Risé P., Porro B., Sala A., Amato M., Tremoli E., Bonomi A., Veglia F. (2021). Effects of Mediterranean Diet or Low-Fat Diet on Blood Fatty Acids in Patients with Coronary Heart Disease. A Randomized Intervention Study. Nutrients.

[96] Tuttolomondo A., Simonetta I., Daidone M., Mogavero A., Ortello A., Pinto A. (2019). Metabolic and Vascular Effect of the Mediterranean Diet. Int. J. Mol. Sci..

[97] Carlos S., De La Fuente-Arrillaga C., Bes-Rastrollo M., Razquin C., Rico-Campà A., Martínez-González M., Ruiz-Canela M. (2018). Mediterranean Diet and Health Outcomes in the SUN Cohort. Nutrients.

[98] Dai J., Lampert R., Wilson P.W., Goldberg J., Ziegler T.R., Vaccarino V. (2010). Mediterranean Dietary Pattern Is Associated with Improved Cardiac Autonomic Function Among Middle-Aged Men: A Twin Study. Circ. Cardiovasc. Qual. Outcomes.

[99] Di Giosia P., Stamerra C.A., Giorgini P., Jamialahamdi T., Butler A.E., Sahebkar A. (2022). The role of nutrition in inflammaging. Ageing Res. Rev..

[100] Kerley C.P. (2019). Dietary patterns and components to prevent and treat heart failure: A comprehensive review of human studies. Nutr. Res. Rev..

[101] Giugliano D., Esposito K. (2005). Mediterranean Diet and Cardiovascular Health. Ann. N. Y. Acad. Sci..

[102] Itsiopoulos C., Mayr H.L., Thomas C.J. (2022). The anti-inflammatory effects of a Mediterranean diet: A review. Curr. Opin. Clin. Nutr. Metab. Care.

[103] Yubero-Serrano E.M., Fernandez-Gandara C., Garcia-Rios A., Rangel-Zuñiga O.A., Gutierrez-Mariscal F.M., Torres-Peña J.D., Marin C., Lopez-Moreno J., Castaño J.P., Delgado-Lista J. (2020). Mediterranean diet and endothelial function in patients with coronary heart disease: An analysis of the CORDIOPREV randomized controlled trial. PLOS Med..

[104] McGrail L., Garelnabi M. (2020). Polyphenolic Compounds and Gut Microbiome in Cardiovascular Diseases. Curr. Pharm. Biotechnol..

[105] Turpin W., Dong M., Sasson G., Raygoza Garay J.A., Espin-Garcia O., Lee S.-H., Neustaeter A., Smith M.I., Leibovitzh H., Guttman D.S. (2022). Mediterranean-Like Dietary Pattern Associations with Gut Microbiome Composition and Subclinical Gastrointestinal Inflammation. Gastroenterology.

[106] Gutiérrez-Díaz I., Fernández-Navarro T., Sánchez B., Margolles A., González S. (2016). Mediterranean diet and faecal microbiota: A transversal study. Food Funct..

[107] Richardson L.A., Izuora K., Basu A. (2022). Mediterranean Diet and Its Association with Cardiovascular Disease Risk Factors: A Scoping Review. Int. J. Environ. Res. Public Health.

[108] Vogel R.A., Corretti M.C., Plotnick G.D. (2000). The postprandial effect of components of the mediterranean diet on endothelial function. J. Am. Coll. Cardiol..

[109] Shannon O.M., Mendes I., KÖchl C., Mazidi M., Ashor A.W., Rubele S., Minihane A.-M., Mathers J.C., Siervo M. (2020). Mediterranean Diet Increases Endothelial Function in Adults: A Systematic Review and Meta-Analysis of Randomized Controlled Trials. J. Nutr..

[110] Mattioli A.V., Palmiero P., Manfrini O., Puddu P.E., Nodari S., Dei Cas A., Mercuro G., Scrutinio D., Palermo P., Sciomer S. (2017). Mediterranean diet impact on cardiovascular diseases: A narrative review. J. Cardiovasc. Med..

[111] Liu Y., Milner M., Klonizakis M. (2018). Physiological effects of a short-term lifestyle intervention based on the Mediterranean diet: Comparison between older and younger healthy, sedentary adults. Nutrition.

[112] Jimenez-Torres J., Alcalá-Diaz J.F., Torres-Peña J.D., Gutierrez-Mariscal F.M., Leon-Acuña A., Gómez-Luna P., Fernández-Gandara C., Quintana-Navarro G.M., Fernandez-Garcia J.C., Perez-Martinez P. (2021). Mediterranean Diet Reduces Atherosclerosis Progression in Coronary Heart Disease: An Analysis of the CORDIOPREV Randomized Controlled Trial. Stroke.

[113] Tosti V., Bertozzi B., Fontana L. (2018). Health Benefits of the Mediterranean Diet: Metabolic and Molecular Mechanisms. J. Gerontol. Ser. A.

[114] Jin Q., Black A., Kales S.N., Vattem D., Ruiz-Canela M., Sotos-Prieto M. (2019). Metabolomics and Microbiomes as Potential Tools to Evaluate the Effects of the Mediterranean Diet. Nutrients.

[115] Tang W.H.W., Li D.Y., Hazen S.L. (2019). Dietary metabolism, the gut microbiome, and heart failure. Nat. Rev. Cardiol..

[116] Drapkina O.M., Ashniev G.A., Zlobovskaya O.A., Yafarova A.A., Dementeva E.V., Kaburova A.N., Meshkov I.O., Sheptulina A.F., Kiselev A.R., Kontsevaya A.V. (2022). Diversities in the Gut Microbial Patterns in Patients with Atherosclerotic Cardiovascular Diseases and Certain Heart Failure Phenotypes. Biomedicines.

[117] Asensio E.M., Ortega-Azorín C., Barragán R., Alvarez-Sala A., Sorlí J.V., Pascual E.C., Fernández-Carrión R., Villamil L.V., Corella D., Coltell O. (2022). Association between Microbiome-Related Human Genetic Variants and Fasting Plasma Glucose in a High-Cardiovascular-Risk Mediterranean Population. Medicina.

[118] Beale A.L., O’Donnell J.A., Nakai M.E., Nanayakkara S., Vizi D., Carter K., Dean E., Ribeiro R.V., Yiallourou S., Carrington M.J. (2021). The Gut Microbiome of Heart Failure with Preserved Ejection Fraction. J. Am. Heart Assoc..

[119] Khan I., Khan I., Jianye Z., Xiaohua Z., Khan M., Hilal M.G., Kakakhel M.A., Mehmood A., Lizhe A., Zhiqiang L. (2022). Exploring blood microbial communities and their influence on human cardiovascular disease. J. Clin. Lab. Anal..

[120] Katsimichas T., Antonopoulos A.S., Katsimichas A., Ohtani T., Sakata Y., Tousoulis D. (2019). The intestinal microbiota and cardiovascular disease. Cardiovasc. Res..

[121] Alam M.J., Puppala V., Uppulapu S.K., Das B., Banerjee S.K. (2022). Human microbiome and cardiovascular diseases. Progress in Molecular Biology and Translational Science.

[122] Masenga S.K., Povia J.P., Lwiindi P.C., Kirabo A. (2023). Recent Advances in Microbiota-Associated Metabolites in Heart Failure. Biomedicines.

[123] Zhou W., Cheng Y., Zhu P., Nasser M.I., Zhang X., Zhao M. (2020). Implication of Gut Microbiota in Cardiovascular Diseases. Oxid. Med. Cell. Longev..

[124] Lv S., Wang Y., Zhang W., Shang H. (2022). Trimethylamine oxide: A potential target for heart failure therapy. Heart.

[125] McMillan A., Hazen S.L. (2019). Gut Microbiota Involvement in Ventricular Remodeling Post–Myocardial Infarction: New Insights into How to Heal a Broken Heart. Circulation.

[126] Zhang W., Dong X.Y., Huang R. (2023). Gut Microbiota in Ischemic Stroke: Role of Gut Bacteria-Derived Metabolites. Transl. Stroke Res..

[127] Suganya K., Son T., Kim K.-W., Koo B.-S. (2021). Impact of gut microbiota: How it could play roles beyond the digestive system on development of cardiovascular and renal diseases. Microb. Pathog..

[128] Kasahara K., Rey F.E. (2019). The emerging role of gut microbial metabolism on cardiovascular disease. Curr. Opin. Microbiol..

[129] Naik S.S., Ramphall S., Rijal S., Prakash V., Ekladios H., Mulayamkuzhiyil Saju J., Mandal N., Kham N.I., Shahid R., Venugopal S. (2022). Association of Gut Microbial Dysbiosis and Hypertension: A Systematic Review. Cureus.

[130] Tousoulis D., Guzik T., Padro T., Duncker D.J., De Luca G., Eringa E., Vavlukis M., Antonopoulos A.S., Katsimichas T., Cenko E. (2022). Mechanisms, therapeutic implications, and methodological challenges of gut microbiota and cardiovascular diseases: A position paper by the ESC Working Group on Coronary Pathophysiology and Microcirculation. Cardiovasc. Res..

[131] Jia Q., Xie Y., Lu C., Zhang A., Lu Y., Lv S., Zhang J. (2019). Endocrine organs of cardiovascular diseases: Gut microbiota. J. Cell. Mol. Med..

[132] Tang W.H.W., Hazen S.L. (2014). The contributory role of gut microbiota in cardiovascular disease. J. Clin. Investig..

[133] Tuteja S., Ferguson J.F. (2019). Gut Microbiome and Response to Cardiovascular Drugs. Circ. Genom. Precis. Med..

[134] MacKay M., Yang B.H., Dursun S.M., Baker G.B. (2023). The Gut-Brain Axis and the Microbiome in Anxiety Disorders, Post-Traumatic Stress Disorder and Obsessive-Compulsive Disorder. Curr. Neuropharmacol..

[135] Chen X., Zhang H., Ren S., Ding Y., Remex N.S., Bhuiyan M.S., Qu J., Tang X. (2023). Gut microbiota and microbiota-derived metabolites in cardiovascular diseases. Chin. Med. J..

[136] Wen Y., Sun Z., Xie S., Hu Z., Lan Q., Sun Y., Yuan L., Zhai C. (2022). Intestinal Flora Derived Metabolites Affect the Occurrence and Development of Cardiovascular Disease. J. Multidiscip. Healthc..

[137] Kelly T.N., Bazzano L.A., Ajami N.J., He H., Zhao J., Petrosino J.F., Correa A., He J. (2016). Gut Microbiome Associates with Lifetime Cardiovascular Disease Risk Profile Among Bogalusa Heart Study Participants. Circ. Res..

[138] Chen L., Ishigami T., Doi H., Arakawa K., Tamura K. (2021). The Types and Proportions of Commensal Microbiota Have a Predictive Value in Coronary Heart Disease. J. Clin. Med..

[139] Lawrence G., Midtervoll I., Samuelsen S.O., Kristoffersen A.K., Enersen M., Håheim L.L. (2022). The blood microbiome and its association to cardiovascular disease mortality: Case-cohort study. BMC Cardiovasc. Disord..

[140] Palmu J., Börschel C.S., Ortega-Alonso A., Markó L., Inouye M., Jousilahti P., Salido R.A., Sanders K., Brennan C., Humphrey G.C. (2023). Gut microbiome and atrial fibrillation—Results from a large population-based study. eBioMedicine.

[141] Wan C., Zhu C., Jin G., Zhu M., Hua J., He Y. (2021). Analysis of Gut Microbiota in Patients with Coronary Artery Disease and Hypertension. Evid. Based Complement. Alternat. Med..

[142] Liao L., Huang J., Zheng J., Ma X., Huang L., Xu W. (2023). Gut microbiota in Chinese and Japanese patients with cardiovascular diseases: A systematic review and meta-analysis. Ann. Saudi Med..

[143] Pevsner-Fischer M., Blacher E., Tatirovsky E., Ben-Dov I.Z., Elinav E. (2017). The gut microbiome and hypertension. Curr. Opin. Nephrol. Hypertens..

[144] Sata Y., Marques F.Z., Kaye D.M. (2020). The Emerging Role of Gut Dysbiosis in Cardio-metabolic Risk Factors for Heart Failure. Curr. Hypertens. Rep..

[145] Dao M.C., Everard A., Aron-Wisnewsky J., Sokolovska N., Prifti E., Verger E.O., Kayser B.D., Levenez F., Chilloux J., Hoyles L. (2016). *Akkermansia muciniphila* and improved metabolic health during a dietary intervention in obesity: Relationship with gut microbiome richness and ecology. Gut.

[146] Marques F.Z., Mackay C.R., Kaye D.M. (2018). Beyond gut feelings: How the gut microbiota regulates blood pressure. Nat. Rev. Cardiol..

[147] Anselmi G., Gagliardi L., Egidi G., Leone S., Gasbarrini A., Miggiano G.A.D., Galiuto L. (2021). Gut Microbiota and Cardiovascular Diseases: A Critical Review. Cardiol. Rev..

[148] Moszak M., Szulińska M., Bogdański P. (2020). You Are What You Eat—The Relationship between Diet, Microbiota, and Metabolic Disorders—A Review. Nutrients.

[149] Kumar T., Dutta R.R., Velagala V.R., Ghosh B., Mudey A. (2022). Analyzing the Complicated Connection Between Intestinal Microbiota and Cardiovascular Diseases. Cureus.

[150] Tuohy K.M., Fava F., Viola R. (2014). ‘The way to a man’s heart is through his gut microbiota’—Dietary pro- and prebiotics for the management of cardiovascular risk. Proc. Nutr. Soc..

[151] Nesci A., Carnuccio C., Ruggieri V., D’Alessandro A., Di Giorgio A., Santoro L., Gasbarrini A., Santoliquido A., Ponziani F.R. (2023). Gut Microbiota and Cardiovascular Disease: Evidence on the Metabolic and Inflammatory Background of a Complex Relationship. Int. J. Mol. Sci..

[152] Tang W.H.W., Hazen S.L. (2017). The Gut Microbiome and Its Role in Cardiovascular Diseases. Circulation.

[153] Özsoy S., Sultanoglu N., Sanlidag T. (2022). The role of mediterranean diet and gut microbiota in type- diabetes mellitus associated with obesity (diabesity). J. Prev. Med. Hyg..

[154] Borton M.A., Shaffer M., Hoyt D.W., Jiang R., Ellenbogen J.B., Purvine S., Nicora C.D., Eder E.K., Wong A.R., Smulian A.G. (2023). Targeted curation of the gut microbial gene content modulating human cardiovascular disease. mBio.

[155] Poli A. (2020). What connection is there between intestinal microbiota and heart disease?. Eur. Heart J. Suppl..

[156] Al-Rubaye H., Perfetti G., Kaski J.-C. (2019). The Role of Microbiota in Cardiovascular Risk: Focus on Trimethylamine Oxide. Curr. Probl. Cardiol..

[157] Hemmati M., Kashanipoor S., Mazaheri P., Alibabaei F., Babaeizad A., Asli S., Mohammadi S., Gorgin A.H., Ghods K., Yousefi B. (2023). Importance of gut microbiota metabolites in the development of cardiovascular diseases (CVD). Life Sci..

[158] Kanitsoraphan C., Rattanawong P., Charoensri S., Senthong V. (2018). Trimethylamine N-Oxide and Risk of Cardiovascular Disease and Mortality. Curr. Nutr. Rep..

[159] Roberts A.B., Gu X., Buffa J.A., Hurd A.G., Wang Z., Zhu W., Gupta N., Skye S.M., Cody D.B., Levison B.S. (2018). Development of a gut microbe–targeted nonlethal therapeutic to inhibit thrombosis potential. Nat. Med..

[160] Palombaro M., Raoul P., Cintoni M., Rinninella E., Pulcini G., Aspromonte N., Ianiro G., Gasbarrini A., Mele M.C. (2022). Impact of Diet on Gut Microbiota Composition and Microbiota-Associated Functions in Heart Failure: A Systematic Review of In Vivo Animal Studies. Metabolites.

[161] Nagatomo Y., Tang W.H.W. (2015). Intersections Between Microbiome and Heart Failure: Revisiting the Gut Hypothesis. J. Card. Fail..

[162] Koay Y.C., Chen Y.-C., Wali J.A., Luk A.W.S., Li M., Doma H., Reimark R., Zaldivia M.T.K., Habtom H.T., Franks A.E. (2021). Plasma levels of trimethylamine-N-oxide can be increased with ‘healthy’ and ‘unhealthy’ diets and do not correlate with the extent of atherosclerosis but with plaque instability. Cardiovasc. Res..

[163] Ringel C., Dittrich J., Gaudl A., Schellong P., Beuchel C.F., Baber R., Beutner F., Teren A., Engel C., Wirkner K. (2021). Association of plasma trimethylamine N-oxide levels with atherosclerotic cardiovascular disease and factors of the metabolic syndrome. Atherosclerosis.

[164] Lee Y., Nemet I., Wang Z., Lai H.T.M., De Oliveira Otto M.C., Lemaitre R.N., Fretts A.M., Sotoodehnia N., Budoff M., DiDonato J.A. (2021). Longitudinal Plasma Measures of Trimethylamine N-Oxide and Risk of Atherosclerotic Cardiovascular Disease Events in Community-Based Older Adults. J. Am. Heart Assoc..

[165] Gencer B., Li X.S., Gurmu Y., Bonaca M.P., Morrow D.A., Cohen M., Bhatt D.L., Steg P.G., Storey R.F., Johanson P. (2020). Gut Microbiota-Dependent Trimethylamine N-oxide and Cardiovascular Outcomes in Patients with Prior Myocardial Infarction: A Nested Case Control Study From the PEGASUS-TIMI 54 Trial. J. Am. Heart Assoc..

[166] Zhou X., Jin M., Liu L., Yu Z., Lu X., Zhang H. (2020). Trimethylamine N-oxide and cardiovascular outcomes in patients with chronic heart failure after myocardial infarction. ESC Heart Fail..

[167] Li W., Huang A., Zhu H., Liu X., Huang X., Huang Y., Cai X., Lu J., Huang Y. (2020). Gut microbiota-derived trimethylamine *N* -oxide is associated with poor prognosis in patients with heart failure. Med. J. Aust..

[168] Chen G., He L., Dou X., Liu T. (2022). Association of Trimethylamine-N-Oxide Levels with Risk of Cardiovascular Disease and Mortality among Elderly Subjects: A Systematic Review and Meta-Analysis. Cardiorenal Med..

[169] Ren H., Zhu B., An Y., Xie F., Wang Y., Tan Y. (2023). Immune communication between the intestinal microbiota and the cardiovascular system. Immunol. Lett..

[170] Bu J., Wang Z. (2018). Cross-Talk between Gut Microbiota and Heart via the Routes of Metabolite and Immunity. Gastroenterol. Res. Pract..

[171] Trøseid M., Andersen G.Ø., Broch K., Hov J.R. (2020). The gut microbiome in coronary artery disease and heart failure: Current knowledge and future directions. eBioMedicine.

[172] Dubinski P., Czarzasta K., Cudnoch-Jedrzejewska A. (2021). The Influence of Gut Microbiota on the Cardiovascular System Under Conditions of Obesity and Chronic Stress. Curr. Hypertens. Rep..

[173] Pastori D., Carnevale R., Nocella C., Novo M., Santulli M., Cammisotto V., Menichelli D., Pignatelli P., Violi F. (2017). Gut-Derived Serum Lipopolysaccharide is Associated with Enhanced Risk of Major Adverse Cardiovascular Events in Atrial Fibrillation: Effect of Adherence to Mediterranean Diet. J. Am. Heart Assoc..

[174] Hernández-Ruiz P., Amezcua-Guerra L.M., López-Vidal Y., González-Pacheco H., Pinto-Cardoso S., Amedei A., Aguirre-García M.M. (2023). Comparative characterization of inflammatory profile and oral microbiome according to an inflammation-based risk score in ST-segment elevation myocardial infarction. Front. Cell. Infect. Microbiol..

[175] Zabell A., Tang W.H.W. (2017). Targeting the Microbiome in Heart Failure. Curr. Treat. Options Cardiovasc. Med..

[176] Kitai T., Tang W.H.W. (2018). Gut microbiota in cardiovascular disease and heart failure. Clin. Sci..

[177] Mamic P., Chaikijurajai T., Tang W.H.W. (2021). Gut microbiome—A potential mediator of pathogenesis in heart failure and its comorbidities: State-of-the-art review. J. Mol. Cell. Cardiol..

[178] Anderson K.M., Ferranti E.P., Alagha E.C., Mykityshyn E., French C.E., Reilly C.M. (2022). The heart and gut relationship: A systematic review of the evaluation of the microbiome and trimethylamine-N-oxide (TMAO) in heart failure. Heart Fail. Rev..

[179] Gallo A., Macerola N., Favuzzi A.M., Nicolazzi M.A., Gasbarrini A., Montalto M. (2022). The Gut in Heart Failure: Current Knowledge and Novel Frontiers. Med. Princ. Pract..

[180] Kimble R., Gouinguenet P., Ashor A., Stewart C., Deighton K., Matu J., Griffiths A., Malcomson F.C., Joel A., Houghton D. (2023). Effects of a mediterranean diet on the gut microbiota and microbial metabolites: A systematic review of randomized controlled trials and observational studies. Crit. Rev. Food Sci. Nutr..

[181] Rejeski J.J., Wilson F.M., Nagpal R., Yadav H., Weinberg R.B. (2022). The Impact of a Mediterranean Diet on the Gut Microbiome in Healthy Human Subjects: A Pilot Study. Digestion.

[182] Pagliai G., Russo E., Niccolai E., Dinu M., Di Pilato V., Magrini A., Bartolucci G., Baldi S., Menicatti M., Giusti B. (2020). Influence of a 3-month low-calorie Mediterranean diet compared to the vegetarian diet on human gut microbiota and SCFA: The CARDIVEG Study. Eur. J. Nutr..

[183] Mitsou E.K., Kakali A., Antonopoulou S., Mountzouris K.C., Yannakoulia M., Panagiotakos D.B., Kyriacou A. (2017). Adherence to the Mediterranean diet is associated with the gut microbiota pattern and gastrointestinal characteristics in an adult population. Br. J. Nutr..

[184] Barber T.M., Kabisch S., Pfeiffer A.F.H., Weickert M.O. (2023). The Effects of the Mediterranean Diet on Health and Gut Microbiota. Nutrients.

[185] Garcia-Mantrana I., Selma-Royo M., Alcantara C., Collado M.C. (2018). Shifts on Gut Microbiota Associated to Mediterranean Diet Adherence and Specific Dietary Intakes on General Adult Population. Front. Microbiol..

[186] Tagliamonte S., Laiola M., Ferracane R., Vitale M., Gallo M.A., Meslier V., Pons N., Ercolini D., Vitaglione P. (2021). Mediterranean diet consumption affects the endocannabinoid system in overweight and obese subjects: Possible links with gut microbiome, insulin resistance and inflammation. Eur. J. Nutr..

[187] Pisanu S., Palmas V., Madau V., Casula E., Deledda A., Cusano R., Uva P., Vascellari S., Boi F., Loviselli A. (2020). Impact of a Moderately Hypocaloric Mediterranean Diet on the Gut Microbiota Composition of Italian Obese Patients. Nutrients.

[188] Ismael S., Silvestre M.P., Vasques M., Araújo J.R., Morais J., Duarte M.I., Pestana D., Faria A., Pereira-Leal J.B., Vaz J. (2021). A Pilot Study on the Metabolic Impact of Mediterranean Diet in Type 2 Diabetes: Is Gut Microbiota the Key?. Nutrients.

[189] García-Montero C., Fraile-Martínez O., Gómez-Lahoz A.M., Pekarek L., Castellanos A.J., Noguerales-Fraguas F., Coca S., Guijarro L.G., García-Honduvilla N., Asúnsolo A. (2021). Nutritional Components in Western Diet Versus Mediterranean Diet at the Gut Microbiota–Immune System Interplay. Implications for Health and Disease. Nutrients.

[190] Gundogdu A., Nalbantoglu O.U. (2023). The role of the Mediterranean diet in modulating the gut microbiome: A review of current evidence. Nutrition.

[191] Rosés C., Cuevas-Sierra A., Quintana S., Riezu-Boj J.I., Martínez J.A., Milagro F.I., Barceló A. (2021). Gut Microbiota Bacterial Species Associated with Mediterranean Diet-Related Food Groups in a Northern Spanish Population. Nutrients.

[192] De Filippis F., Pellegrini N., Vannini L., Jeffery I.B., La Storia A., Laghi L., Serrazanetti D.I., Di Cagno R., Ferrocino I., Lazzi C. (2016). High-level adherence to a Mediterranean diet beneficially impacts the gut microbiota and associated metabolome. Gut.

[193] Clark J.S., Simpson B.S., Murphy K.J. (2022). The role of a Mediterranean diet and physical activity in decreasing age-related inflammation through modulation of the gut microbiota composition. Br. J. Nutr..

[194] Muralitharan R.R., Jama H.A., Xie L., Peh A., Snelson M., Marques F.Z. (2020). Microbial Peer Pressure: The Role of the Gut Microbiota in Hypertension and Its Complications. Hypertension.

[195] García-López M., Martínez-González M., Basterra-Gortari F., Barrio-López M., Gea A., Beunza J. (2014). Adherence to the Mediterranean dietary pattern and heart rate in the SUN project. Eur. J. Prev. Cardiol..

[196] Yntema T., Koonen D.P.Y., Kuipers F. (2023). Emerging Roles of Gut Microbial Modulation of Bile Acid Composition in the Etiology of Cardiovascular Diseases. Nutrients.

[197] Bailey M.A., Holscher H.D. (2018). Microbiome-Mediated Effects of the Mediterranean Diet on Inflammation. Adv. Nutr..

